# Hypoxia-induced circWSB1 promotes breast cancer progression through destabilizing p53 by interacting with USP10

**DOI:** 10.1186/s12943-022-01567-z

**Published:** 2022-03-29

**Authors:** Rui Yang, Hang Chen, Lei Xing, Bin Wang, Mengting Hu, Xiaoqiang Ou, Hong Chen, Yumei Deng, Dawei Liu, Rong Jiang, Junxia Chen

**Affiliations:** 1 Department of Cell Biology and Genetics, Chongqing Medical University, #1 Yixueyuan Road, Chongqing, 400016 China; 2 Department of Endocrine and Breast Surgery, The First Affiliated Hospital of Chongqing Medical University, #1 Yixueyuan Road, Chongqing, 400016 China; 3 Department of Anesthesiology, Yongchuan Hospital of Chongqing Medical University, #439 XuanHua Road, Chongqing, 402160 China; 4 Laboratory of Stem Cells and Tissue Engineering, Chongqing Medical University, #1 Yixueyuan Road, Chongqing, 400016 China

**Keywords:** Hypoxia, circWSB1, Breast cancer, p53, USP10

## Abstract

**Background:**

Hypoxia has long been considered as a hallmark of solid tumors and is closely associated with tumor progression. Circular RNAs (circRNAs) have been identified as a critical modulator in various cancers. However, the connections between hypoxia and circRNAs are largely unknown.

**Methods:**

Here, we investigated the expression profile of circRNAs in breast cancer (BC) MCF-7 cells under hypoxia and normoxia using microarray. We identified a novel hypoxia-responsive circRNA named circWSB1, whose expression pattern, potential diagnostic value and prognostic significance were assessed by qRT-PCR and in situ hybridization. Loss- and gain-of-function investigations in vivo and in vitro were performed to determine the biological functions of circWSB1. Mechanistically, chromatin immunoprecipitation and dual luciferase reporter assays were carried out to analyze the biogenesis of circWSB1. Furthermore, biotin-labeled RNA pull-down, mass spectrometry, RNA immunoprecipitation, fluorescent in situ hybridization, RNA electrophoretic mobility shift, deletion-mapping, co-immunoprecipitation assays and rescue experiments were applied to investigate the interaction between circWSB1 and Ubiquitin-specific peptidase 10 (USP10) as well as the relationship between USP10 and p53.

**Results:**

We found that the expression of circWSB1 was significantly upregulated in BC tissues and correlated with poor clinical outcomes, which might serve as an independent prognostic factor for BC patients. Ectopic expression of circWSB1 promoted the proliferation of BC cell in vitro and in vivo. Mechanistically, circWSB1 was transcriptionally upregulated by HIF1α in response to hypoxia and could competitively bind to deubiquitinase USP10 to prevent the access of p53 to USP10 in BC cells, leading to degradation of p53 and tumor progression of BC.

**Conclusions:**

Taken together, our findings disclose a novel mechanism that hypoxia-inducible circWSB1 could interact with USP10 to attenuate USP10 mediated p53 stabilization and promote the progression of BC, providing an alternative prognostic biomarker and therapeutic target for BC.

**Supplementary Information:**

The online version contains supplementary material available at 10.1186/s12943-022-01567-z.

## Introduction

Breast cancer is the most frequent malignancy among women worldwide, accounting for about 30% of all cases of cancer and 15% of all cancer deaths in females [1, 2]. Although great progress has been achieved in early diagnosis, surgery, chemoradiotherapy, endocrine therapy and targeted therapy over the past decades, BC is still the leading cause of cancer death among women in more than 100 countries around the world [3]. Thus, it is urgent to find novel biomarkers and therapeutic targets to diagnose and treat BC.

Hypoxia is commonly observed in BC as well as many other solid tumors, which usually resulted from excessive proliferation of tumor cells and aberrant disorganization of tumor vasculature [4]. In BC, hypoxia is associated with aggressiveness, drug resistance and poor prognosis [5]. Hypoxia-inducible factor 1 (HIF1) is considered as the central regulator of cellular response to hypoxia [6]. HIF1 activates the transcription of a diverse genes through combining with the hypoxia response elements (HREs) within their promoter regions to assist cancer cells to adapt to hypoxic conditions, which leads to a more aggressive and metastatic phenotype of cancer cells [4, 7]. Increased expression of HIF1α has been observed in BC and high level of HIF1α is an independent predictor of mortality of patients with BC [8]. However, the molecular mechanism of HIF1 mediated hypoxic response in BC is still not fully understood.

Circular RNAs (circRNAs) are a type of single-stranded RNA transcripts with no 5′ cap or 3′ poly A tail but a covalently closed loop structure generated from pre-mRNAs through back-splicing [9]. Some circRNAs have been identified in eukaryotes with a cell- or tissue-specific expression pattern as well as high stability and evolutionary conservation [10]. Mounting evidence indicated that circRNAs participate in the pathological process of many human diseases, especially cancer [11]. Recently, several circRNAs have been reported to be closely related to hypoxia and play crucial roles in cancer progression. For instance, circDENND4C is overexpressed in breast cancer and its expression is induced by hypoxia. Knocking down circDENND4C can suppress migration, invasion and glycolysis of BC cells through up-regulation of miR-200b/c under hypoxia [12]. Another research demonstrated that hypoxia-related circDENND2A facilitates glioma invasiveness via sponging miR-625-5p [13]. Moreover, Ou et al. reported that hypoxia induces the expression of circ-0000977 and the circ-0000977/miR-153/HIF1A and ADAM10 axis may regulate immune escape in pancreatic cancer cells [14]. However, the biogenesis and biological functions of most circRNAs in cancers under hypoxia remain largely unknown.

The p53 is the tumor suppressor with the highest correlation with human tumors. p53 acts as a transcription factor and regulates various cellular functions, such as cell cycle, apoptosis, cell differentiation, cell aging, DNA repair and energy metabolism [15]. Recent study revealed that *TP53* derived circTP53 was also involved in the proliferation of tumor cells [16]. p53 signaling pathway inactivation plays a significant role in pathogenesis of BC. Approximate 75% of BC patients are wild type p53 [17]. Besides gene deletion and point mutation, abnormal post-translational modification of proteins has been proved to be an alternative mechanism for p53 inactivation. The regulation of p53 activity and stability is mainly carried out through post-translational modification such as phosphorylation, ubiquitination and acetylation. MDM2 E3 ubiquitin ligase-mediated ubiquitination of p53 induces p53 degradation and plays a main role in regulation of p53, while some deubiquitinases (DUBs) can reverse ubiquitination of p53 and stabilize its protein level [18, 19]. It was found that the deubiquitinase USP10 could deubiquitinate and stabilize p53 and suppress MDM2-mediated p53 ubiquitination and degradation [20]. Moreover, another deubiquitinating enzyme USP7 has also been proved to deubiquitinate and strongly stabilize p53 [21]. However, the additional p53 regulatory mechanism and its role in the pathogenesis of BC remain enigmatic.

Here, we identified a novel circRNA derived from the exon 3, 4, 5 and 6 of *WSB1* gene, termed as circWSB1, which was upregulated in BC tissues and closely correlated with unfavorable clinical prognosis of BC patients. We found that circWSB1 was transcriptionally upregulated by HIF1α under hypoxic conditions and promoted the proliferation of BC cells in vivo and in vitro. The mechanism research demonstrated that circWSB1 could directly bind to deubiquitinase USP10 and abate USP10 mediated p53 stabilization, leading to the degradation of p53 and progression of BC. Our findings provide novel insights into the underlying molecular mechanism of how hypoxia-induced circWSB1 contributes to the progression of BC, revealing the possibility of circWSB1 to serve as an alternative therapeutic target and prognostic marker for BC.

## Materials and methods

### Cell culture

Human BC cell lines (MDA-MB-231, MCF-7, SKBR-3, MDA-MB-453 and BT-549) and normal mammary epithelial cell line MCF-10A were obtained from American Type Culture Collection (ATCC) (Manassas, VA, USA). MCF-7, MDA-MB-231 and MDA-MB-453 cells were cultured in DMEM medium (Gibco, Carlsbad, CA, USA), BT-549 and SKBR-3 cells were maintained in RPMI-1640 medium (Gibco), supplemented with 10% fetal bovine serum, 100 mg/mL streptomycin and 100 U/mL penicillin. MEBM BulletKit (Lonza, Basel, Switzerland) was used to culture MCF-10A cells. All these cells were maintained in a humidified incubator at 37 °C with 5% CO_2_. As to hypoxic treatment, cells were cultured in a tri‑gas incubator with 1% O_2_, 94% N_2_ and 5% CO_2_.

### RNA isolation and microarray analysis

The total RNA of MCF-7 cells cultured under normoxia or hypoxia was exacted using Trizol reagent (Takara, Dalian, China) and quantified by Nanodrop 2000 spectrophotometer (Thermo Fisher Scientific, Waltham, MA, USA). The integrity of the obtained RNA was determined by Agilent Bioanalyzer 2100 (Agilent Technologies, CA, USA). The Agilent Human ceRNA MicroArray 2019 (Agilent Technologies) was employed to analyze the differentially expressed circRNAs and mRNAs in MCF-7 cells treated with normoxia and hypoxia. The Agilent SurePrint G3 Human Gene Expression v3 Microarray was applied to analyze the differentially expressed genes after circWSB1 knockdown. Data analysis were performed by OE Biotechnology Co., Ltd., (Shanghai, China).

### Analysis of TCGA data

The RNA-seq data of 1100 breast cancer tissues and 111 para-cancerous tissues and corresponding clinical information were downloaded from The Cancer Genome Atlas (TCGA) data portal (https://portal.gdc.cancer.gov/). The expression level of HIF1α was quantified by the TPM (transcripts per kilobase per million) of each sample.

### Tissues specimens

The 100 pairs of BC tissues and non-tumorous tissues were acquired from the patients who were diagnosed with BC at the First Affiliated Hospital of Chongqing Medical University (Chongqing, China). None of these patients had received preoperative chemotherapy or radiotherapy. Tissue samples were stored in liquid nitrogen before RNA extraction.

### qRT-PCR

The total RNA was synthesized into cDNA with PrimeScript RT Reagent Kit (Takara) in accordance with the manufacturer’s protocols. The cDNA was amplified with TB Green Premix Ex Taq (Takara) on a Bio-Rad CFX96 system (Bio-Rad, CA, USA). The expression of circRNA and mRNA was determined by 2^–ΔΔCT^ and normalized by β-actin. The primers used in the study were listed in Table S1.

### RNA in situ hybridization (ISH)

ISH was conducted with a digoxin-labeled probe specific for circWSB1 (Digoxin-5’- TCTTGGTGCCATGCAAGAGACCAAATTCCATCAGAATGTC-3’-Digoxin) to evaluate the expression of circWSB1 on tissue microarrays (Outdo Biotech, Shanghai, China) which contained 288 BC tissues and 123 normal tissues. Briefly, the tissue microarrays were dewaxed and rehydrated, then digested with proteinase K and followed by hybridization with above mentioned circWSB1 probe at 45 °C overnight. After that, the tissues were incubated with biotin-conjugated antibodies against digoxin at 4 °C overnight, then stained with DAB. The expression of circWSB1 was quantified by multiplying the scores of the intensity of positive staining (strong = 3, moderate = 2, weak = 1 and negative = 0) and the percentage of positive-stained cells (> 76% = 4, 51–75% = 3, 26–50% = 2, 5–25% = 1, < 5% = 0). The samples were defined as low or high expression groups by the mean of ISH scores.

### Plasmids, siRNAs and cell transfection

The full length of liner sequence of circWSB1 was amplified and subcloned into the lentiviral vector pLC5-ciR (Geneseed, Guangzhou, China) to construct circWSB1 overexpression vector, termed as pLC5-circWSB1. Two siRNAs targeting the back-splicing site of circWSB1 and negative control were synthesized by Geneseed. Full-length of human USP10 cDNA and its truncations were amplified and subcloned into pcDNA3.1–3 × Flag (RiboBio, Guangzhou, China). Human HIF1α cDNA was amplified and subcloned into pcDNA3.1 (RiboBio) to establish HIF1α overexpression plasmids. siRNAs targeting USP10 or HIF1α were synthesized by RiboBio. Cell transfection was conducted with Lipofectamine 2000 (Invitrogen, Carlsbad, CA, USA) following the manufacturer’s instructions.

### Cell proliferation, cell cycle and apoptosis assays

The growth curves of BC cells were obtained using Cell Counting Kit-8 (Bosterbio, Wuhan, China) according to the protocols of manufacturer. As to colony formation assays, BC cells (2000/well) were inoculated into 6-well plates and cultured for two weeks, followed by fixing and staining with 0.5% crystal violet. Cell cycle and apoptosis assays were analyzed on a flow cytometer (Becon Dickinson FACSCalibur, NY, USA) with PI staining and Dead Cell Apoptosis Kit (Thermo Fisher Scientific), respectively.

### Fluorescence in situ hybridization (FISH) and Immunofluorescence (IF) co-staining

The coverslips seeded with BC cells were incubated with antibodies specific for USP10 (1:100, Abcam, Burlingame, CA, USA) at 4 °C overnight and FITC-conjugated secondary antibodies at 37 °C for 1 h. Then, the coverslips were hybridized with Cy3-labeled probes (5’-Cy3-TGCAAGAGACCAAATTCCATCAG-Cy3-3’) (Geneseed) targeting the junction site of circWSB1 using Fluorescent In Situ Hybridization kit (RiboBio) according to the manufacturer’s protocols, followed by counterstaining with DAPI.

### Cytoplasmic/Nuclear fractionation

The cytoplasmic and nuclear fractionation was conducted with PARIS™ Kit (Thermo Fisher Scientific). The RNA of each fraction was isolated according to the recommended protocols, followed by reverse transcription and qRT-PCR.

### Dual-luciferase reporter assay

Human *WSB1* promoter containing four presumptive HREs and corresponding mutants were subcloned into pGL3-Basic (Genecreate, Wuhan, China) to construct luciferase reporter vectors. Cells expressing HIF1α were co-transfected with HRE-WT or HRE-MT reporter constructs and renilla luciferase plasmids. The cells were maintained under hypoxic or normoxic conditions for about 48 h. The luciferase activity of the reporters was detected with Dual Luciferase Reporter Assay Kit (Hanbio, Shanghai, China) and normalized to renilla luciferase activity.

### Chromatin immunoprecipitation (ChIP)

Chromatin immunoprecipitation was performed with antibodies specific for HIF1α (CST, Beverly, MA, USA), IgG control (CST) and SimpleChIP® Enzymatic Chromatin IP Kit (CST) in accordance with the manufacturer’s instructions.

### RNA pull-down and mass spectrometry

The biotin-labeled probes targeting the junction site of circWSB1 (5’-TGGTGCCATGCAAGAGACCAAATTCCATCAGAATG-3’-Biotin) were synthesized using MAXIscript™ Kit (Thermo Fisher Scientific) following the manufacturer’s protocols. RNA pull-down assay was carried out with Pierce™ Magnetic RNA–Protein Pull-Down Kit (Thermo Fisher Scientific) following the manufacturer’s instructions. The retrieved proteins were detected by western blot or mass spectrometry analysis at Genecreate.

### Co-immunoprecipitation (Co-IP)

Co-immunoprecipitation was executed with antibodies specific for USP10 (Abcam) or p53 (CST), IgG control (CST) and Pierce™ Classic Magnetic IP/Co-IP Kit (Thermo Fisher Scientific). In Brief, cells were harvested and lysed with IP lysis/wash buffer supplemented with protease inhibitor cocktail for 20 min on ice, then centrifugated at 14,000 g for 20 min. The supernatant was collected and incubated with antibodies (5 μg) on a rotator at 4 °C overnight. After that, 25 μL Protein A/G Magnetic Beads were pre-washed and incubated with the lysate/antibody mix for 4 h at 4 °C. The beads were collected with a magnetic stand and then washed with IP lysis/wash buffer and ultra-pure water. The proteins were eluted with 100 μL of Lane Marker Sample Buffer and heated at 100 °C for 10 min, then followed by western blot.

### RNA immunoprecipitation (RIP)

RNA immunoprecipitation assay was performed with antibodies specific for USP10 (Abcam), IgG control (CST) and RNA Immunoprecipitation Kit (Geneseed) according to the recommended conditions. The co-precipitated RNAs and USP10 proteins were detected with qRT-PCR and western blot, respectively.

### RNA electrophoretic mobility shift assay (EMSA)

The biotin-labeled probes (probe 1, 5’-UACCAAUUCAAGCAGUUUAAGAUUGCCAAGACAAAAUAGUGAUGGUGGUCAG-3’-Biotin, probe 2, 5’-AGAAAAAUAAGCCUCGUGAACAUAUUAUAGACUGUGGAGAUAUAGUCUGGAGU-3’- Biotin) were prepared as described above. RNA EMSA was conducted with LightShift® Chemiluminescent RNA EMSA Kit (Thermo Fisher Scientific) in accordance with the manufacturer’s protocols.

### Lentivirus packaging and infection

The full-length of USP10 cDNA was subcloned into CMV-MCS-EF1-P2A lentiviral vector. shRNA targeting circWSB1 or USP10 was designed on the basis of si-circ#1 or si-USP10#1 and inserted into U6-MCS-PGK-EGFP or U6-CMV-P2A-BSD lentiviral vectors (Hanbio), respectively. To obtain lentivirus, the lentiviral vectors were co-transfected with packaging plasmid psPAX2 and pMD2G into 293 T cells. Viral supernatants were collected at 48 h and 72 h after transfection, followed by ultracentrifugation at 82700 g for 2 h. Cell were infected with the obtained lentivirus and selected with puromycin or blasticidin to establish stable cells.

### Xenograft mouse model

The four-week-old female BALB/c nude mice were purchased from Tengxin Biotechnology Co., LTD (Chongqing, China) and housed under the standard conditions at the Center of Experimental Animals of Chongqing Medical University. MCF-7 cells (1 × 10^7^) were subcutaneously inoculated into the dorsal flanks of the randomly grouped nude mice. The tumor size of each mouse was monitored and calculated by length × width^2^ × 0.5. Four weeks later, the tumor-bearing mice were sacrificed and the xenografts were imaged with small animal imaging system, then excised and weighed. Survival analysis was performed in parallel with the above tumor growth studies.

### Immunohistochemistry

Paraffin-embedded sections were dewaxed and rehydrated, then incubated with primary antibodies specific for Ki67 (1:100, CST) or p53 (1:100, CST) at 4 °C overnight and biotin-labeled secondary antibodies at 37 °C for 1 h. The slides were then stained with DAB and hematoxylin, followed by photographing under a microscope (Leica, Wetzlar, Germany).

### Western blot analysis

Total proteins of BC cells were extracted with RIPA lysis buffer containing PMSF and subjected to SDS-PAGE, then transferred onto PVDF membranes (Millipore, Billerica, MA, USA). The membranes were blocked with 5% skimmed milk and incubated with primary antibodies specific for HIF1α (1:2000, CST), USP10 (1:2000, Abcam), p53 (1:2000, CST), Bax (1:2000, CST), p21 (1:2000, CST) or β-actin (1:5000, CST) at 4 °C overnight and HRP-conjugated secondary antibodies (1:5000, CST) at room temperature for 2 h. The bands were finally visualized using SuperSignal™ West Femto Maximum Sensitivity Substrate (Thermo Fisher Scientific).

### Statistical analysis

Statistical analyses were performed with SPSS 21.0 (IBM, SPSS, Chicago, IL, USA). Student’s t-test and one-way ANOVA were applied in the comparison of differences between two or three groups, respectively. The relevance between groups were examined by χ^2^ test. Receiver operating characteristic (ROC) curve was used to assess the diagnostic value of circWSB1 in BC. Survival and recurrence curves were plotted by Kaplan–Meier method and assessed by log-rank test. Multivariate Cox proportional hazards regression models were applied to analyze the risk factors for the overall survival of BC patients. *P* < 0.05 was regarded as statistically significant.

## Results

### CircWSB1 is identified in BC cells under hypoxia

**Fig. 1:**
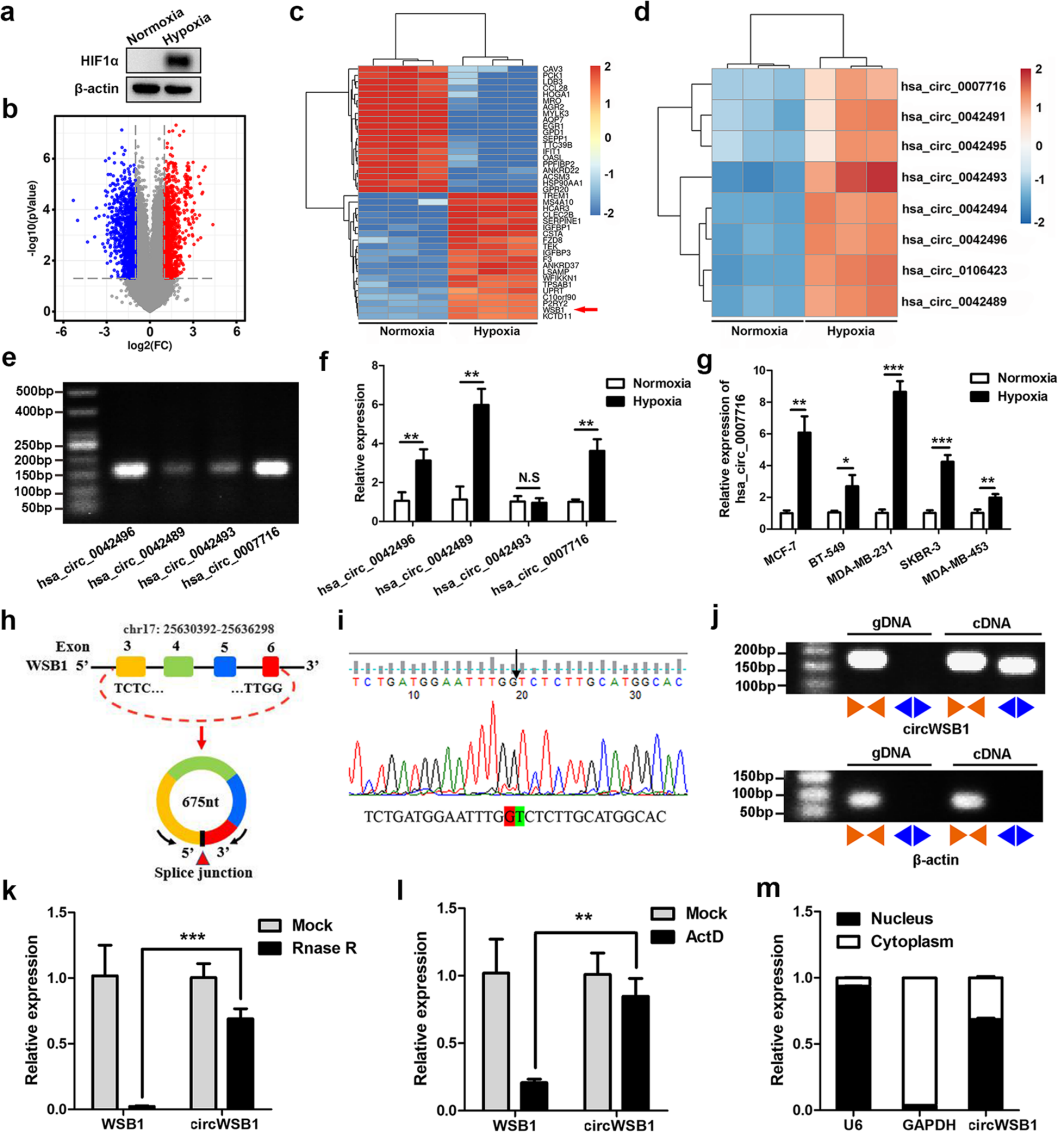
Identification of circWSB1 in BC cells in response to hypoxia. **a** Western blot analysis of HIF1α in MCF-7 cells cultured under normoxia or hypoxia for 48 h. **b** Volcano plot of the differentially expressed circRNAs between MCF-7 cells treated with normoxia and hypoxia for 48 h. The blue dots and red dots represent downregulated and upregulated circRNAs with statistical significance, respectively. **c** and **d** Heatmaps of 20 most increased and decreased mRNAs (**c**) and eight circRNAs derived from *WSB1* (**d**) between normoxic and hypoxic MCF-7 cells. **e** RT-PCR products of hsa_circ_0042496, hsa_circ_0042489, hsa_circ_0042493 and hsa_circ_0007716 detected by agarose gel electrophoresis. **f** qRT-PCR analyses of the relative expression of hsa_circ_0042496, hsa_circ_0042489, hsa_circ_0042493 and hsa_circ_0007716 in MCF-7 cells cultured under normoxia and hypoxia. **g** Relative expression of hsa_circ_0007716 detected by qRT-PCR in multiple BC cell lines treated with normoxia and hypoxia. **h** Schematic diagram illustrating the generation of hsa_circ_0007716 from its host gene *WSB1*. **i** Sanger sequencing of back-splicing junction of hsa_circ_0007716 (circWSB1). **j** circWSB1 and β-actin were amplified from gDNA and cDNA of MCF-7 cells using both convergent and divergent primers, respectively, followed by agarose gel electrophoresis. **k** qRT-PCR analysis to determine the relative level of circWSB1 and WSB1 mRNA in MCF-7 cells treated with or without Rnase R at 37 ℃ for 30 min. **l** Relative expression of circWSB1 and WSB1 mRNA in MCF-7 cells treated with or without actinomycin D (5 μg/mL) was examined by qRT-PCR. **m** Cytoplasmic/Nuclear fractionation followed by qRT-PCR indicating the subcellular localization of circWSB1 in MCF-7 cells cultured under hypoxia. Data are shown as mean ± SD and representative of three independent experiments in (**f-g**) and (**k-m**). *P < 0.05, **P < 0.01, ***P < 0.001

In order to understand whether hypoxia is present in BC, we initially analyzed the expression of HIF1α in 1100 BC tissues and 111 normal tissues of TCGA database. It was found that HIF1α was significantly increased in BC tissues and its level was negatively associated with the overall survival of BC patients (Fig. S1a and b), indicating that hypoxia is indeed a hallmark in the progression of BC. To gain insight into the hypoxia-associated circRNAs in BC, we analyzed the circRNA and mRNA expression profiles in MCF-7 cells cultured under normoxia and hypoxia (1% O_2_, 48 h), finding that 974 circRNAs and 732 mRNAs were upregulated, while 1366 circRNAs and 1530 mRNAs were downregulated in MCF-7 cells under hypoxia with a cut-off criteria of fold change ≥ 2.0 and *P* < 0.05 (Fig. 1a and b and Fig. S1c). KEGG pathway analysis was performed with these differentially expressed mRNAs to reveal the significantly enriched signaling pathways (Fig. S1d). Among these differentially expressed circRNAs and mRNAs, we found that WSB1 and eight circRNAs derived from it were all significantly increased in hypoxic MCF-7 cells (Fig. 1c and d). Previous work showed that WSB1 was induced by hypoxic conditions and could promote the invasion and metastasis of cancer [22]. Hence, we were interested in how these eight *WSB1*-derived circRNAs exert their functions in BC under hypoxia. We detected whether these circRNAs were present in MCF-7 cells by RT-PCR and agarose electrophoresis. As depicted in Fig. 1e, only four of these circRNAs (hsa_circ_0042493, hsa_circ_0042496, hsa_circ_0042489 and hsa_circ_0007716) could be amplified with divergent primers in MCF-7 cells, of which hsa_circ_0007716 was the most abundant one. These four circRNAs were further detected by qRT-PCR in MCF-7 cells treated with normoxia or hypoxia. As a result, hsa_circ_0042489 was the most upregulated circRNA under hypoxia, followed by hsa_circ_0007716 and hsa_circ_0042496 (Fig. 1f). Considering the expression abundance and fold changes under hypoxia, we finally chose hsa_circ_0007716 for the further studies. To validate the former results, qRT-PCR analyses were expanded to multiple BC cell lines treated with normoxia and hypoxia, revealing that hsa_circ_0007716 was also substantially increased in all of these cell lines (MCF-7, BT-549, MDA-MB-231, SKBR-3 and MDA-MB-453) exposed to hypoxia (Fig. 1g).

To make clear of the origin of hsa_circ_0007716, we searched UCSC Genome Browser (https://genome.ucsc.edu/index.html) and found that hsa_circ_0007716 was a circular transcript generated from the back-splicing of the exon 3, 4, 5 and 6 of *WSB1* gene located on human chromosome 17 with a length of 675 nt, which was preliminarily confirmed by sanger sequencing (Fig. 1h and i). Therefore, we firstly named hsa_circ_0007716 as circWSB1. Next, the covalent closed-loop structure of endogenous circWSB1 was verified by RT-PCR with convergent and divergent primers, along with β-actin as a negative control. The results indicated that only circWSB1, rather than β-actin, could be amplified with divergent primers in cDNA (Fig. 1j). Furthermore, circWSB1 was resistant to the degradation of RNase R, while WSB1 mRNA was degraded (Fig. 1k). To further verify the stability of circWSB1, we treated MCF-7 cells with actinomycin D, an inhibitor of RNA synthesis, and found that circWSB1 was much more stable than WSB1 mRNA (Fig. 1l). Cytoplasmic/nuclear fractionation assay followed by qRT-PCR indicated that circWSB1 was dominantly located in the nucleus of hypoxic MCF-7 cells (Fig. 1m). These findings led us to conclude that circWSB1 is a *bona fide* circRNA.

### HIF-1α increases the production of circWSB1 in response to hypoxia

**Fig. 2:**
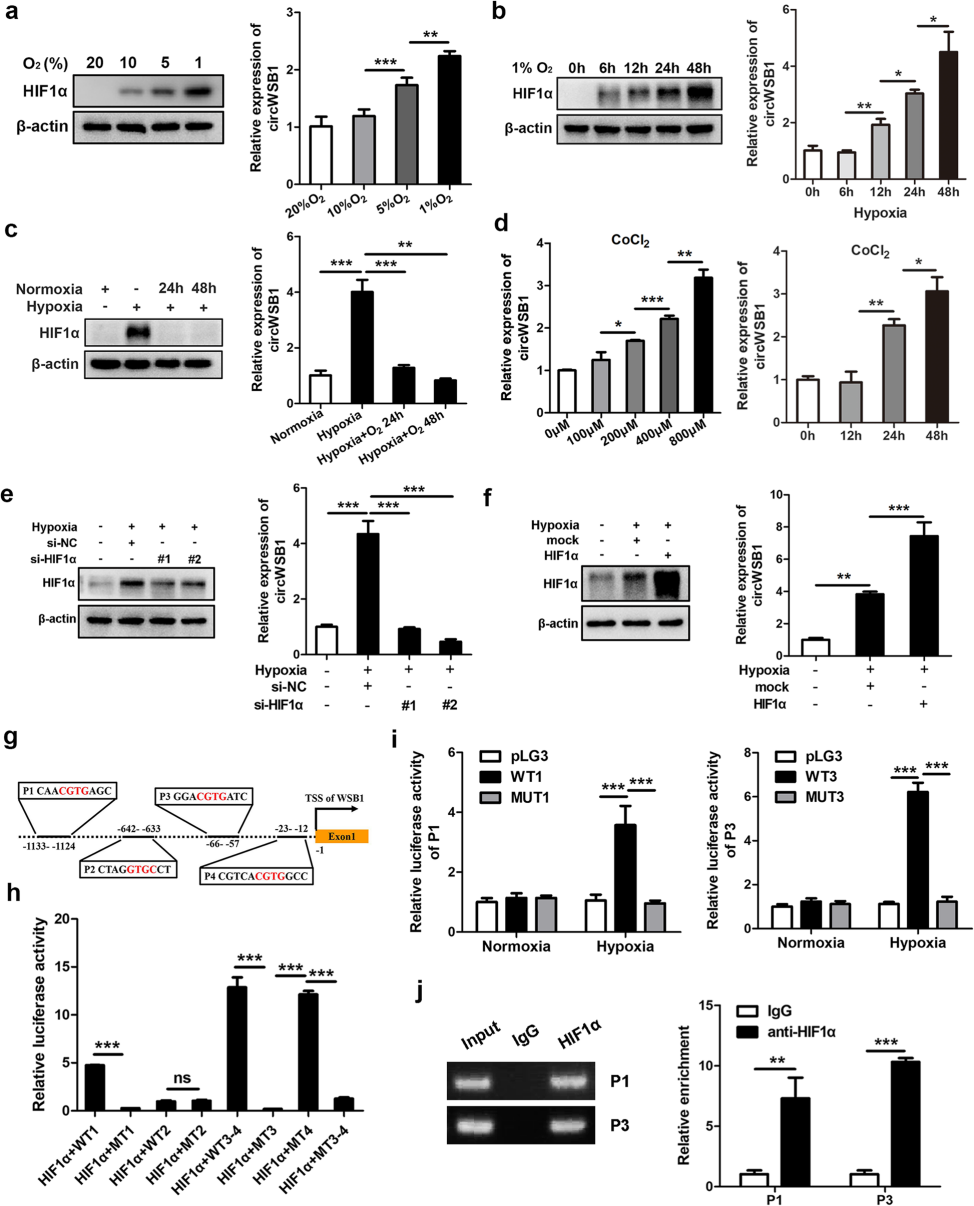
HIF-1α combines with *WSB1* promoter to increase the expression of circWSB1 under hypoxia. **a** and **b** Western blot and qRT-PCR analyses of HIF1α protein level and circWSB1 expression in MCF-7 cells treated with indicated concentration of O_2_ for 24 h (**a**) or different periods of time under 1% O_2_ (**b**). **c** Western blot and qRT-PCR analyses showing the HIF1α protein level and circWSB1 expression in MCF-7 cells cultured under indicated conditions. **d** qRT-PCR analyses to examine the relative expression of circWSB1 in MCF-7 cells treated with different concentration of CoCl_2_ or 400 μM CoCl_2_ for indicated periods of time. **e** and **f** Western blot and qRT-PCR analyses of the HIF1α protein level and circWSB1 expression in MCF-7 cells after knockdown (**e**) or overexpression (**f**) of HIF1α. **g** Schematic illustration of putative HREs in the promoter region of *WSB1* gene. **h** and **i** Luciferase reporter assays for the relative activity of the putative HREs of *WSB1* in MCF-7 cells treated with indicated conditions. **j** ChIP assays were performed with anti-HIF1α or IgG control in MCF-7 cells cultured under hypoxia. Data are shown as mean ± SD and representative of three independent experiments in (**a-f**) and (**h-j**). *P < 0.05, **P < 0.01, ***P < 0.001

To further characterize how circWSB1 was induced by hypoxia, we treated MCF-7 cells with different concentrations of O_2_ and different periods of time under hypoxia (1% O_2_). qRT-PCR analyses revealed that circWSB1 was induced by hypoxia in a concentration- and time-dependent manner (Fig. 2a and b). Moreover, the expression of circWSB1 was restored to baseline after transferring the cells from hypoxia to normoxic conditions (Fig. 2c). Additionally, treatment of MCF-7 cells with CoCl_2_, a hypoxic mimetic reagent to induce a cellular pseudo-hypoxia by stabilize HIF1α, could also lead to an increase of circWSB1 in a dose- and time-dependent manner (Fig. 2d). These results make us speculate whether circWSB1 was regulated by HIF1α under hypoxic circumstances. To test this hypothesis, we constructed overexpression plasmids and siRNAs of HIF1α to overexpress or knockdown of HIF1α, respectively (Fig. S2). Intriguingly, knockdown of HIF1α led to a significant reduction of circWSB1 under hypoxia (Fig. 2e). In contrast, ectopic expression of HIF1α elevated the level of circWSB1 (Fig. 2f). Next, Ensembl (http://asia.ensembl.org/index.html) and JASPAR (http://jaspar.genereg.net/) database were used to seek for the promoter of *WSB1* and analyze the binding motifs for transcription factor HIF1α, respectively. As depicted in Fig. 2g, four putative HREs were found within the promoter region of *WSB1* gene. Consequently, dual-luciferase reporter assays indicated that transfection of HIF1α overexpression plasmid significantly increased the luciferase activity of the *WSB1* promoters containing the HIF1α binding sites P1 and P3, rather than P2 or P4 (Fig. 2h). Nevertheless, these HIF1α-induced luciferase activity of the reporters was only increased under hypoxia, but not normoxic conditions (Fig. 2i). ChIP assays further verified the direct binding of HIF1α with the sites P1 and P3 of the *WSB1* promoter under hypoxia (Fig. 2j). These data demonstrated that the expression of circWSB1 was driven by HIF1α under hypoxic conditions.

### CircWSB1 is highly expressed in BC and correlated with patient prognosis

**Fig. 3:**
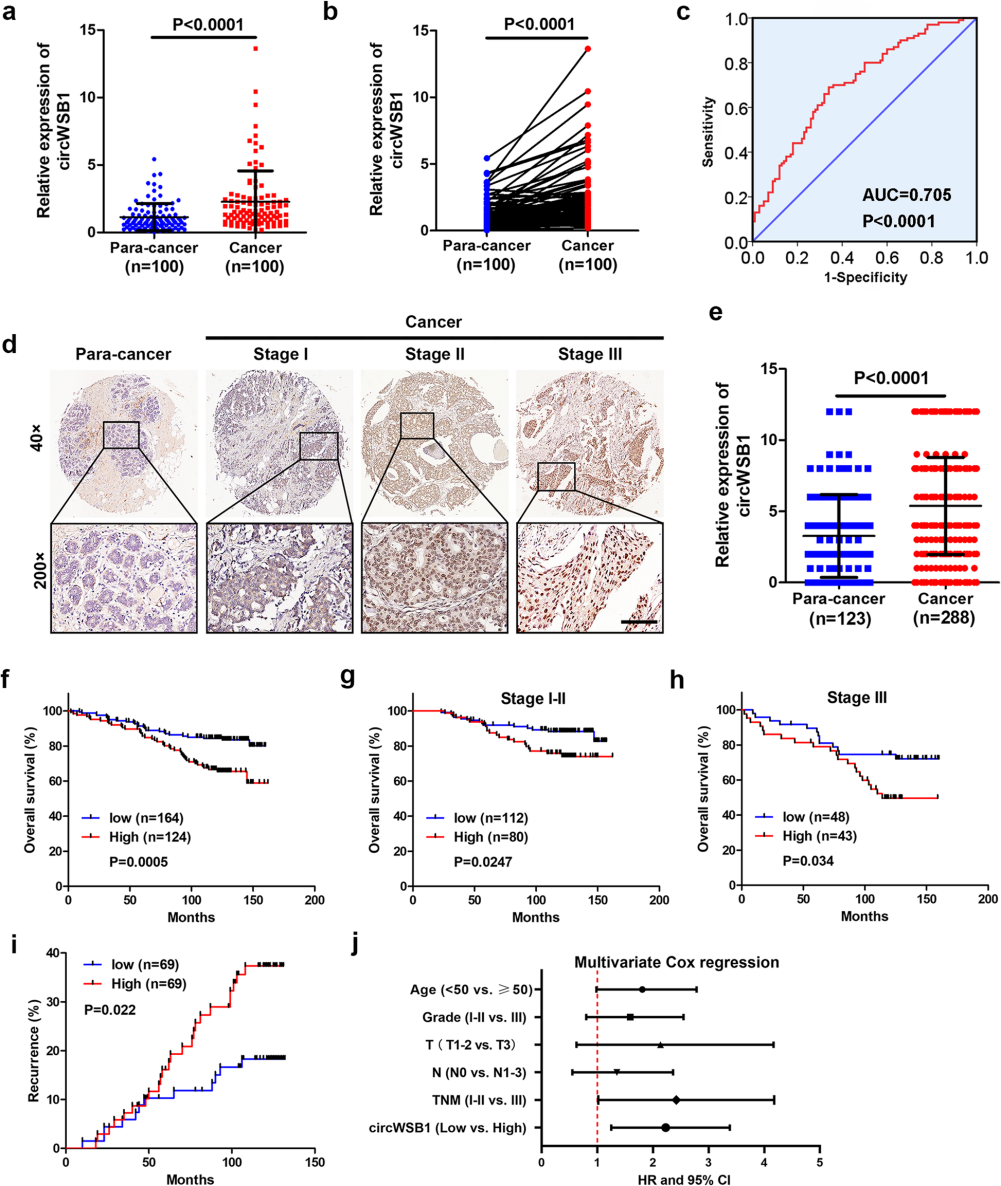
CircWSB1 is upregulated in BC and related to poor prognosis.** a** and** b** Relative expression of circWSB1 in 100 pairs of BC and para-cancerous tissues. **c** ROC curve to assess the diagnostic value of circWSB1 in BC. **d** Representative images of ISH staining of circWSB1 on TMAs. Scale bar, 200 μm. **e** Quantification of the ISH score of circWSB1 on TMAs. **f**–**h** Kaplan–Meier survival curves of BC patients according to the circWSB1 level. BC patients were categorized into circWSB1 low and high expression group by the mean of circWSB1 expression. **i** Recurrence analysis of BC patients. **j** Multivariate Cox regression analysis of risk factors correlated with overall survival of BC patients

In an effort to get knowledge of the expression pattern and clinical significance of circWSB1 in BC, qRT-PCR was applied to detect the expression level of circWSB1 in a cohort of 100 pairs of clinical specimens containing BC tissues and adjacent non-tumorous tissues, which indicated that circWSB1 was markedly increased in tumor samples (Fig. 3a and b). ROC curve demonstrated that the level of circWSB1 could effectively distinguish BC from non-tumor tissues and the sensitivity and specificity were 0.70 and 0.64 if the cut off value was 1.07 (Fig. 3c). The relationship between the expression level of circWSB1 and the clinicopathological characteristics of these BC patients was analyzed and listed in Table 1. The expression of circWSB1 was significantly correlated with T stage of BC patients. We next examined the expression of circWSB1 using ISH in another cohort on TAMs which containing 288 paraffin-embedded BC tissues and 123 para-cancerous tissues, finding that circWSB1 was substantially upregulated in BC tissues (Fig. 3d and e). The correlation between circWSB1 expression and clinicopathological features of the 288 BC patients was listed in Table S2. Kaplan–Meier survival analysis was further applied to compare the prognosis of BC patients grouped by the mean of ISH score of circWSB1, indicating that patients with high level of circWSB1 had worse overall survival than those with low circWSB1 level (Fig. 3f). Analogous results were observed in BC patients in stage I-II and stage III (Fig. 3g and h). Moreover, recurrence analysis uncovered that the expression level of circWSB1 was positively associated with the recurrence rate of BC patients (Fig. 3i). Furthermore, multivariate analysis indicated that circWSB1 could be a standalone risk factor for BC patients (Fig. 3j). Together, these findings suggested that circWSB1 was significantly upregulated in BC and might be a prognostic marker for BC patients.

**Table 1:** The relationship between circWSB1 expression and the clinicopathological characteristics of 100 BC patients

Characteristics		circWSB1		Chi-square	*P* value
		Low	High		
**Age**	< 50	23	20	0.367	0.545
	≥ 50	27	30		
**Grade**	I-II	38	31	2.291	0.130
	III	12	19		
**T stage**	T1-2	42	33	4.320	0.038*
	T3-4	8	17		
**N stage**	N0	29	35	1.563	0.211
	N1-3	21	15		
**TNM stage**	I-II	44	37	3.184	0.074
	III	6	13		

^*^, *P* < 0.05

### CircWSB1 promotes the viability of BC cells in vitro and in vivo

**Fig. 4:**
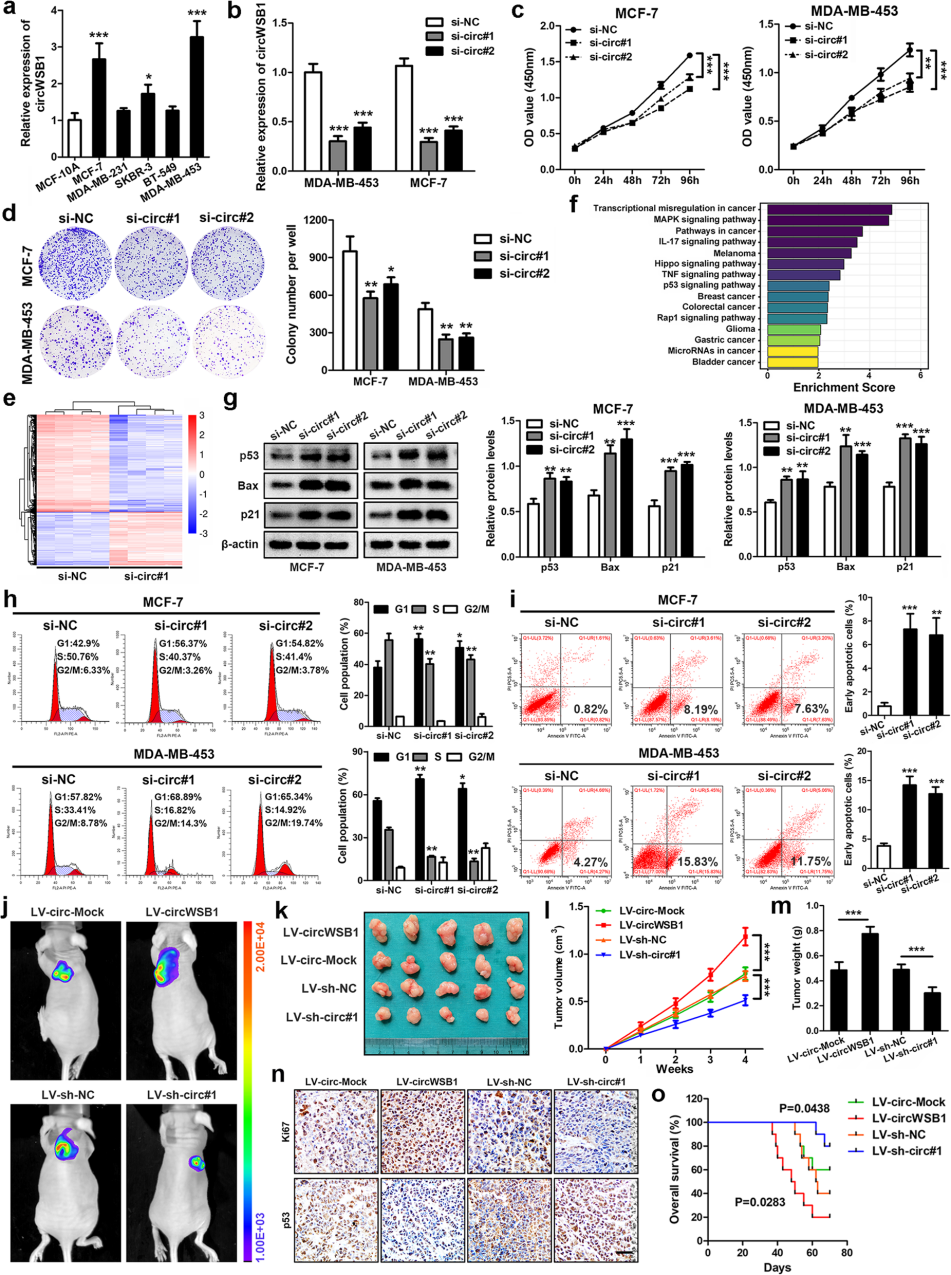
CircWSB1 promotes the proliferation of BC cells in vitro and in vivo. **a** Relative expression of circWSB1 in BC cell lines and normal breast epithelial cell MCF-10A was detected by qRT-PCR. **b** qRT-PCR analysis of circWSB1 expression in MCF-7 and MDA-MB-453 cells transfected with indicated siRNAs. **c** and **d** CCK-8 (**c**) and colony formation assays (**d**) were conducted in hypoxic MCF-7 and MDA-MB-453 cells after circWSB1 depletion. **e** Heatmap of differentially expressed mRNAs after knockdown of circWSB1 in MCF-7 cells under hypoxia. **f** KEGG pathway analysis of differentially expressed mRNAs as per (**e**). **g** Western blot analyses of MCF-7 and MDA-MB-453 cells after depletion of circWSB1 with indicated antibodies. **h** and **i** Cell cycle progression (**h**) and apoptosis rate (**i**) of hypoxic MCF-7 and MDA-MB-453 cells transfected with indicated siRNAs were analyzed by flow cytometry. **j** Representative bioluminescence imaging of tumor-bearing mice inoculated with indicated stable MCF-7 cells. **k** Photograph of xenograft tumors removed from each nude mouse (*n* = 5). **l** Growth curves of xenograft tumors of each group of nude mice were minored and measured once a week. **m** Tumor weight was calculated. **n** IHC staining to analyze the level of Ki67 and p53 in xenograft tumors, scale bar, 100 μm. **o** Kaplan–Meier survival curves of the tumor-bearing mice (*n* = 10). Data are shown as mean ± SD and representative of three independent experiments in (**a-d**) and (**g-i**). *P < 0.05, **P < 0.01, ***P < 0.001

To study the possible function of circWSB1 in BC, we firstly detected the expression of circWSB1 in a panel of BC cell lines (MCF-7, BT-549, SKBR-3, MDA-MB-231 and MDA-MB-453). Compared with the normal breast epithelial cell line MCF-10A, the expression of circWSB1 was substantially increased in MCF-7 and MDA-MB-453 cells (Fig. 4a), which were selected for the further investigations. Then, two siRNAs targeting the back-splicing site of circWSB1 and overexpression vector were constructed to knockdown or overexpress circWSB1, which were verified by qRT-PCR in MFC-7 and MDA-MB-453 cells (Fig. 4b and Fig. S3a). Importantly, the overexpression and knockdown system did not affect the expression of WSB1 mRNA and protein levels (Fig. S3 b and c). CCK-8 growth curves indicated that knockdown of circWSB1 in MCF-7 and MDA-MB-453 cells led to significant inhibition of cell viability under hypoxia (Fig. 4c). On the contrary, enforced expression of circWSB1 markedly promoted the proliferation of hypoxic MCF-7 and MDA-MB-453 cells (Fig. S3d). In addition, colony formation assays further demonstrated that downregulation of circWSB1 impaired the growth of MCF-7 and MDA-MB-453 cells under hypoxia, whereas overexpression of circWSB1 exhibited an entirely opposite effect (Fig. 4d and Fig. S3e**)**. To uncover the underlying signaling pathways involved in the effect of circWSB1 on the growth of BC cells, we analyzed the differentially expressed genes (DEGs) using microarray in MCF-7 cells transfected with siRNAs (si-circ#1) and negative control (si-NC) under hypoxia, finding 1085 upregulated and 2090 downregulated genes (fold change ≥ 1.5 and *P* < 0.05) after knockdown of circWSB1 (Fig. 4e). KEGG pathway analysis showed that circWSB1 was involved in many cell-growth-related pathways (Fig. 4f). Among them, p53 signaling pathway attracted our attention since p53 has been a well-known tumor suppressor with the highest correlation with human tumors up to now [23]. To validate whether circWSB1 exerted its function through p53 signaling pathway, western blot was performed and indicated that knockdown of circWSB1 not only increased the protein level of p53 but also its downstream targets p21 and Bax (Fig. 4g), whereas overexpression of circWSB1 produced an opposite effect on the expression of these proteins (Fig. S3f). The effects of circWSB1 on p53 signaling pathway raised the possibility that circWSB1 modulates the cell cycle and apoptosis of BC cells. As expected, circWSB1 deficiency triggered cell cycle arrest at G1 phase in MCF-7 and MDA-MB-453 cells exposed to hypoxic conditions (Fig. 4h). Furthermore, depletion of circWSB1 resulted in an increased apoptosis rate of hypoxic MCF-7 and MDA-MB-453 cells (Fig. 4i). These results suggested that circWSB1 could promote BC cell proliferation under hypoxia through p53 signaling pathway.

To investigate the effect of circWSB1 on BC cell growth in vivo, MCF-7 cells stably overexpressing or knocking down of circWSB1 and their controls were constructed and verified by qRT-PCR (Fig. S3g). These stable MCF-7 cells were then subcutaneously inoculated into the dorsal flanks of four-week-old female BALB/c nude mice to establish BC xenograft models. Four weeks later, we found that enforced expression of circWSB1 substantially enhanced the growth of the xenograft tumors, whereas loss of circWSB1 significantly retarded the tumor growth in vivo (Fig. 4j-m). IHC staining assays indicated that the tumors derived from circWSB1 overexpression group had higher level of proliferation index Ki67 and lower expression of p53, while depletion of circWSB1 exhibited an opposite effect (Fig. 4n). In addition, survival analyses suggested that circWSB1 expression was negatively correlated with the survival time of tumor-bearing mice (Fig. 4o). These findings demonstrated that circWSB1 played an oncogenic role in the progression of BC.

### CircWSB1 directly binds to deubiquitinase USP10

**Fig. 5:**
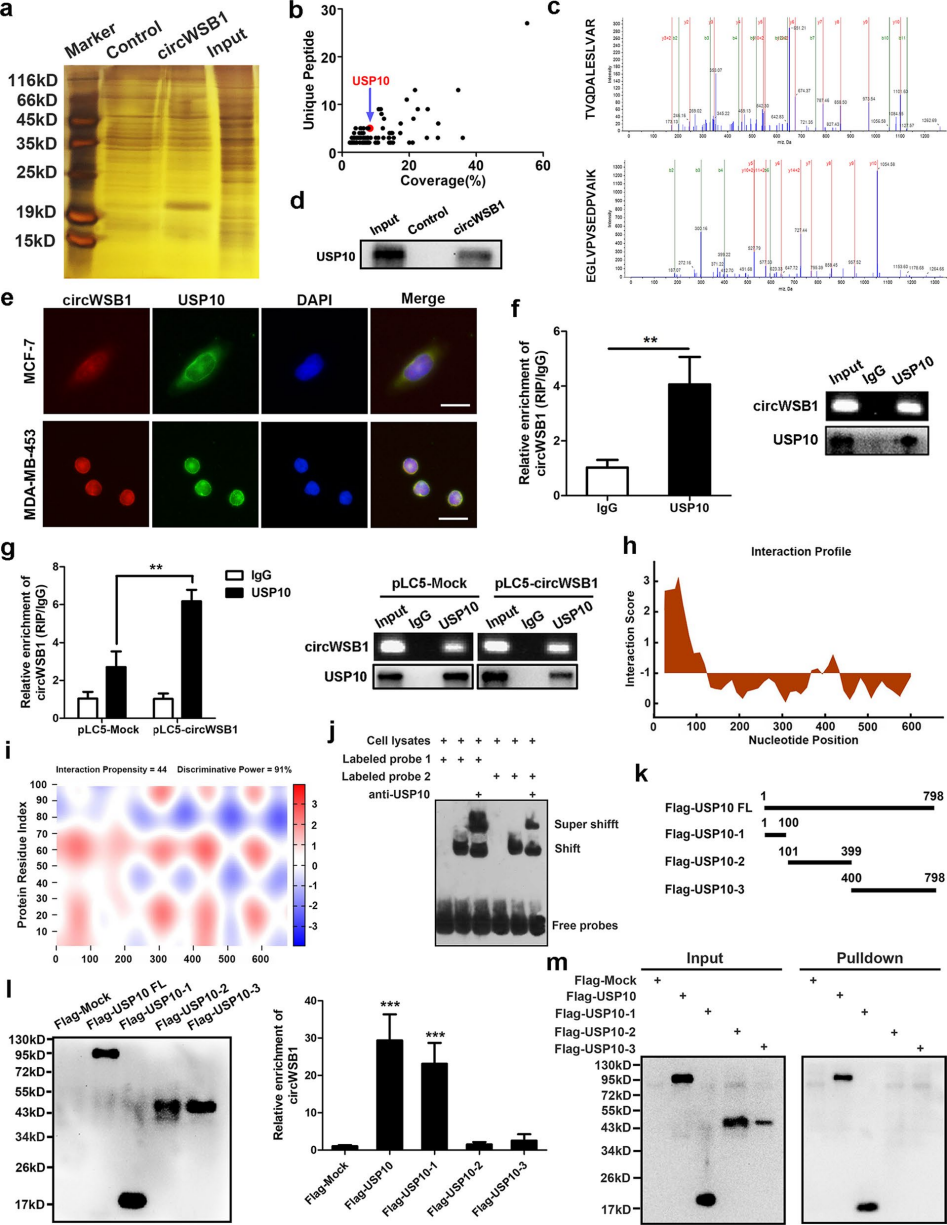
CircWSB1 directly binds with deubiquitinase USP10. **a** Silver staining of proteins pulled down by biotin-labeled probe specific for circWSB1 and control probe. **b** The RNA pull-down proteins identified by mass spectrometry analysis. **c** Unique peptides of USP10 identified by mass spectrometry analysis. **d** The interaction between circWSB1 and USP10 was verified by western blot. **e** FISH and IF co-staining indicating the co-localization of circWSB1 (red) and USP10 (green) in MCF-7 and MDA-MB-453 cells cultured under hypoxia. Scale bar, 50 μm. **f** and **g** RIP assays were carried out in hypoxic MCF-7 cells under indicated conditions using anti-USP10 and IgG control, followed by qRT-PCR. **h** Interaction profile of circWSB1 on USP10. **i** Heatmap of interaction between circWSB1 and N-terminal region (1-100aa) of USP10. **j** RNA EMSA showing the interaction between USP10 protein and biotin-labeled probe of circWSB1. **k** Schematic diagram of full-length and truncated USP10 protein. **l** RIP assays were executed with anti-Flag in hypoxic MCF-7 cells transfected with indicated full-length or truncated USP10 plasmids with 3 × Flag. Co-precipitated proteins and RNAs were purified and followed by western blot and qRT-PCR, respectively. **m** RNA pull-down assays using biotin-labeled circWSB1 probe in hypoxic MCF-7 cells expressing full-length of USP10 and its deletion mutants. The pulled down proteins were subjected to western blot. Data are shown as mean ± SD and representative of three independent experiments in (**f-g**) and **i**. **P < 0.01, ***P < 0.001

The aforementioned findings urged us to explore how circWSB1 affects p53 in BC. Therefore, RNA pull-down assay was performed with a biotin-labeled probe targeting the back-splicing site of circWSB1 in hypoxic MCF-7 cells, followed by mass spectrometry (Fig. 5a). With the cut-off criteria of unique peptides ≥ 1 and confidence ≥ 95%, we identified that 141 unique proteins were pulled down by probes specific for circWSB1, but not negative control probe (Fig. 5b). Although p53 was not observed among these proteins, strikingly, we found that the deubiquitinase USP10 was pulled down by circWSB1, with two peptides identified by mass spectrometry analysis (Fig. 5c). It has been documented that USP10 binds with and deubiquitinates p53, reversing MDM2-induced ubiquitination and proteasomal degradation of p53 [20]. Thus, it made us to suppose that circWSB1 might regulate p53 level through USP10 under hypoxia. To further validate the result of mass spectrometry analysis, western blot was applied and confirmed the interaction between circWBS1 and USP10 (Fig. 5d). FISH and IF co-staining assay revealed that circWSB1 and USP10 were co-located in both cytoplasm and nucleus of MCF-7 and MDA-MB-453 cells, providing evidence for their interaction (Fig. 5e). Consequently, the interaction between circWSB1 and USP10 was further substantiated by RIP assay using antibodies specific for USP10 (Fig. 5f). Moreover, ectopic expression of circWSB1 led to an increased enrichment of circWSB1 by USP10 (Fig. 5g**)**.

To probe the structural basis of the interactions between circWSB1 and USP10, the online database catRAPID (http://s.tartaglialab.com/page/catrapid_group) was used to analyze the precise interaction between these two molecules. Interestingly, there was a high affinity between the two fragments (25-75nt and 76-126nt) of circWSB1 and the N-terminal region (1-100aa) of USP10 with a relative high propensity and discriminative power (Fig. 5h and i). On this basis, RNA EMSA was carried out with two biotin-labeled fragments (25-75nt and 76-126nt) of circWSB1 used as probes, confirming that both of them were essential for the interaction between circWSB1 and USP10 (Fig. 5j). To validate the circWSB1 binding region on USP10, we constructed Flag-tagged full-length USP10 and its deletion mutants according to the USP10 function domains (Fig. 5k). Subsequent RIP assay showed that the N-terminal region (1-100aa) of USP10, but not other domains, was crucial for its interaction with circWSB1 (Fig. 5l). Furthermore, the direct interaction between endogenous circWSB1 and the N-terminal region (1-100aa) of USP10 was detected by RNA pull-down assay (Fig. 5m). These results confirmed the direct binding between circWSB1 and deubiquitinase USP10. Notably, enforced expression or knockdown of circWSB1 affect neither the transcript nor protein level of USP10 (Fig. S4a and b). On the contrary, manipulation of USP10 also had no effect on the expression of circWSB1 (Fig. S4c). Therefore, our data suggested circRNA could physically interact with USP10.

### USP10 stabilizes and deubiquitinates p53 and inhibits BC cell proliferation

**Fig. 6:**
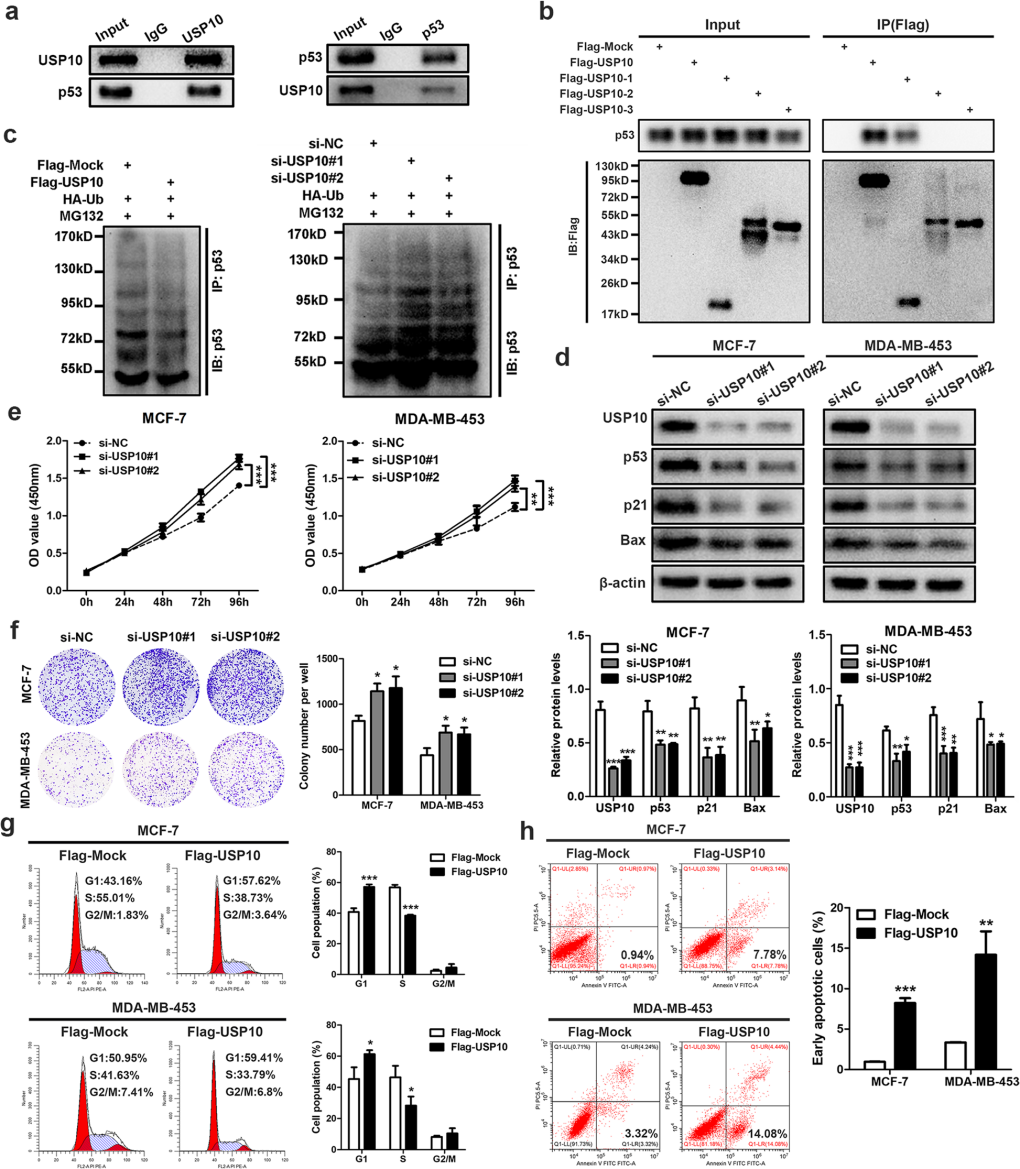
USP10 interacts with p53 and suppresses BC cell growth. **a** Co-IP assays to analyze the direct interaction between USP10 and p53 in MCF-7 cells using anti-USP10 or anti-p53, respectively. **b** Co-IP was applied in MCF-7 cells transfected with indicated full-length or truncated USP10 plasmids using anti-Flag. **c** Ubiquitination of p53 was analyzed in MG132-treated MCF-7 cells bearing Flag-Mock and Flag-USP10 or si-NC, si-USP10#1 and si-USP10#2. **d** Western blot analyses of hypoxic MCF-7 and MDA-MB-453 cells after knockdown of USP10 with indicated antibodies. **e** and **f** CCK-8 (**e**) and colony formation assays (**f**) were conducted in MCF-7 and MDA-MB-453 cells transfected with indicated siRNAs under hypoxia. **g** and **h** Cell cycle progression (**g**) and apoptosis rate (**h**) of hypoxic MCF-7 and MDA-MB-453 cells were analyzed by flow cytometry after enforced expression of USP10. Data are showed as mean ± SD and representative of three independent experiments in (**e–h**). *P < 0.05, **P < 0.01, ***P < 0.001

It has been previously reported that USP10 could bind with and stabilize p53 in renal cell carcinoma cells [20]. To ascertain whether this combination exists in BC, we performed Co-IP assays in MCF-7 cells under hypoxia and found that USP10 and p53 were coimmunoprecipitated with each other (Fig. 6a). Deletion-mapping investigations with Flag-tagged full-length USP10 and its truncated mutants revealed that the N-terminal region (1-100aa) of USP10 was critical for the p53 interaction (Fig. 6b). Then we detected the effect of USP10 on p53 ubiquitination in hypoxic MCF-7 cells. As depicted in Fig. 6c, p53 ubiquitination was diminished by upregulation of USP10, however, depletion of USP10 enhanced ubiquitination of p53. Moreover, the protein levels of p53 and its downstream targets p21 and Bax were markedly increased when USP10 was overexpressed, while downregulation of USP10 produced an opposite effect (Fig. S5a and Fig. 6d). Next, we examined the effect of USP10 on the proliferation, cell cycle and apoptosis of BC cells. The cell growth curves indicated that overexpression or silencing of USP10 attenuated and enchanced the proliferation of hypoxic MCF-7 and MDA-MB-453 cells, respectively (Fig. S5b and Fig. 6e). Similar results were observed in colony formation assays (Fig. S5c and Fig. 6f). Furthermore, flow cytometry analysis showed that enforced expression of USP10 led to cell cycle arrest at G1 phase and apoptosis under hypoxic conditions (Fig. 6g and h). Collectively, these data demonstrated that USP10 could deubiquitinate and stabilize p53, thereby inhibiting the growth of BC cells under hypoxia.

### CircWSB1 promotes BC cell growth by disrupting USP10-p53 interaction.

**Fig. 7:**
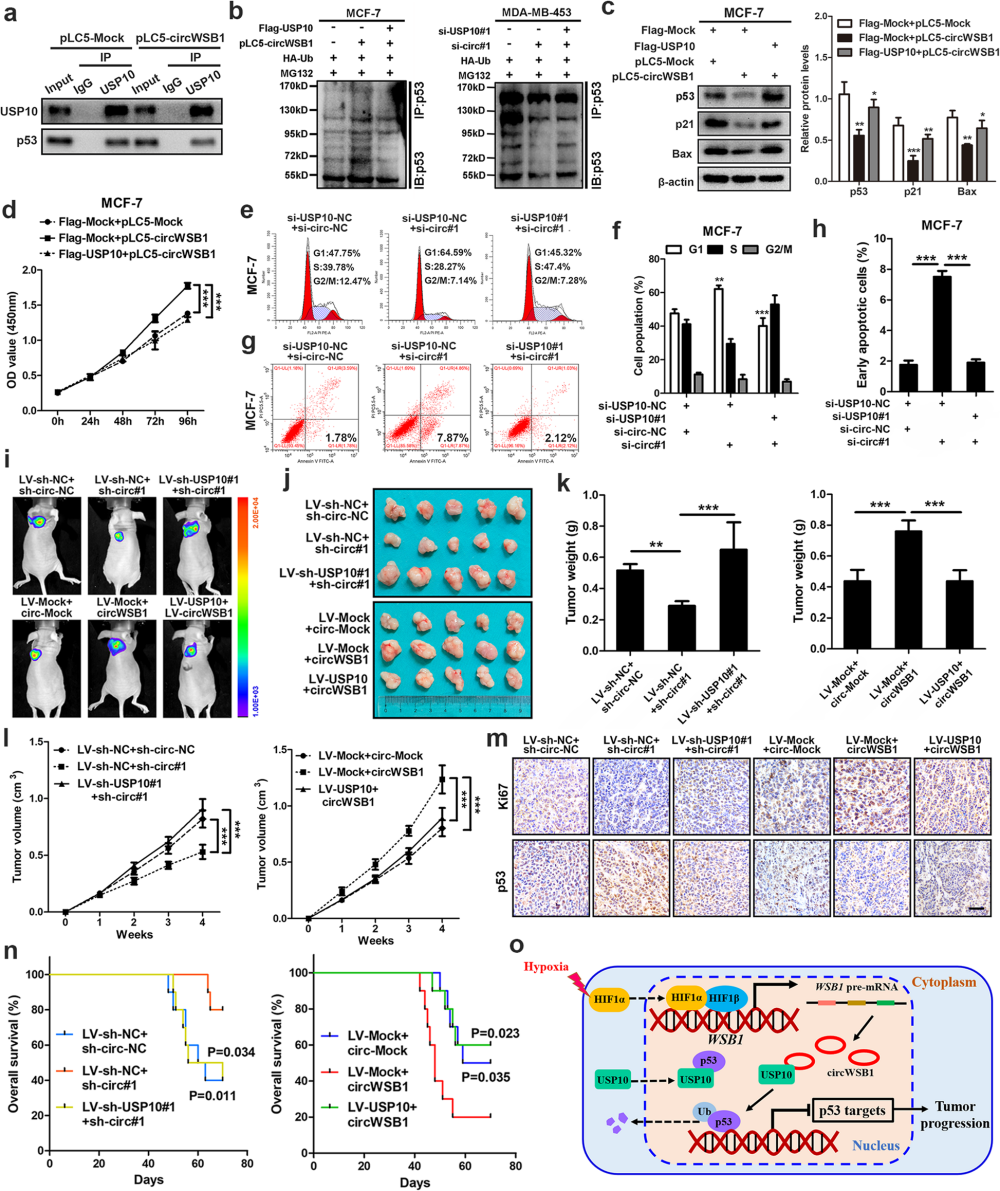
CircWSB1 facilitates BC cell growth through inhibiting the interaction between USP10 and p53. **a** Co-IP assays were conducted with anti-USP10 in hypoxic MCF-7 cells transfected with indicated plasmids. **b** Ubiquitination levels of p53 were detected in MG132-treated MCF-7 and MDA-MB-453 cells co-transfected with indicated vectors or siRNAs under hypoxia. **c** Western blot analyses of MCF-7 cells after transfected with indicated vectors under hypoxia. **d** CCK-8 assays of MCF-7 cells under hypoxic conditions. **e–h** Flow cytometric cell cycle assays (**e, f**) and apoptosis analyses (**g, h**) of hypoxic MCF-7 cells treated with indicated siRNAs. **i** Representative bioluminescence imaging of tumor-bearing mice injected with indicated stable MCF-7 cells. **j** Photograph of xenograft tumors removed from each nude mouse (*n* = 5). **k** The weight of each group of xenograft tumor was calculated. **l** Growth curves of xenograft tumors of each group of nude mice were minored and measured once a week. **m** IHC staining to determine the expression of Ki67 and p53 in xenograft tumors, scale bar, 100 μm. **n** Kaplan–Meier survival curves of the tumor-bearing mice (*n* = 10). **o** Schematic diagram illustrating the generation of circWSB1 and the mechanisms how circWSB1 promotes the progression of BC under hypoxia. Data are showed as mean ± SD or representative of three independent experiments in (**c-h**). **P < 0.01, ***P < 0.001

Previous experiments demonstrated that both circWSB1 and p53 protein could bind with the N-terminal region (1-100aa) of USP10. Therefore, we infer that circWSB1 probably exerted its function by disrupting the interaction between USP10 and p53. To test this hypothesis, we performed Co-IP assays in MCF-7 cells with anti-USP10, finding that overexpression of circWSB1 significantly reduced the amount of p53 enriched by USP10, which suggested that circWSB1 impeded the combination of USP10 and p53 (Fig. 7a). Further ubiquitination assays indicated that the poly-ubiquitination of p53 induced by overexpression of circWSB1 could be reversed by enforced expression of USP10, meanwhile, the decrease of p53 poly-ubiquitination produced by circWSB1 depletion could be rescued by silencing of USP10 (Fig. 7b). Moreover, western blot revealed that overexpression or knockdown of circWSB1 inhibited or enhanced the protein level of p53 as well as p21 and Bax in BC cells, while these effects could be abolished by enforced expression or silencing of USP10, respectively (Fig. 7c and Fig. S6a).

We then examined the functional relationship between circWSB1 and USP10 in BC cells. CCK-8 and colony formation assays showed that the proliferation-promoting effect induced by overexpression of circWSB1 could be rescued by upregulation of USP10 in MCF-7 cells, whereas the proliferation-suppressing effect produced by circWSB1 depletion could be reversed by knockdown of USP10 in MDA-MB-453 cells under hypoxia (Fig. 7d and Fig. S6b and c). Similarly, flow cytometry analyses indicated that the cell cycle arrest and apoptosis induced by knockdown of circWSB1 could be rescued by silencing of USP10 in hypoxic MCF-7 and MDA-MB-453 cells (Fig. 7e-h and Fig. S6d and e). To further validate above results, we observed the reciprocal effect between circWSB1 and USP10 in vivo. Unequivocally, enforced expression or knockdown of USP10 could attenuate the tumor-promoting or inhibiting effects induced by overexpression or silencing of circWSB1, respectively (Fig. 7i-l). Moreover, IHC staining indicated that ectopic expression of circWSB1 led to an increase of Ki67 and decrease of p53 in xenograft tumors, knockdown of circWSB1 produced the opposite, while overexpression or depletion of USP10 reversed these effects (Fig. 7m). Further survival analysis of tumor-bearing mice in parallel with the above experiments revealed that the decreased or increased overall survival rates induced by ectopic expression or silencing of circWSB1 could be reversed by enforced expression or knockdown of USP10, respectively. (Fig. 7n). These results suggested that hypoxia-induced circWSB1 directly bound with USP10 and disrupted the interaction between USP10 and p53, leading to the degradation of p53 and progression of BC.

## Discussion

It has already been established that regional hypoxia is one of the most common mircoenvironmental properties of solid tumors, which contributes to the malignant progression, recurrence, distant metastasis and resistance to chemoradiotherapy in many human cancers by modulating the expression of numerous protein-coding genes as well as non-coding RNAs [24, 25]. Recently, a growing number of lncRNAs and miRNAs have been reported to be involved in the process of cell adaption to hypoxia through regulating the expression of HIF1α and its targets or being modulated by HIF1α under hypoxic circumstances [26, 27]. However, whether circRNA plays critical roles in those hypoxia-mediated malignant behaviors in cancers remains elusive, especially in BC. In the present study, we identified a novel hypoxia-responsive circRNA generated form *WSB1* gene, termed as circWSB1, which was shown to be transcriptionally modulated by HIF1α under hypoxic conditions. Of note, the expression of circWSB1 was substantially elevated in the majority of BC tissues and high level of circWSB1 might be an independent prognostic factor for the overall survival of BC patients. Moreover, enforced expression of circWSB1 enhanced the viability of BC cells under hypoxia in vitro. Furthermore, the xenograft mouse model further confirmed the tumor-promoting effect of circWSB1 in vivo. Our findings revealed the oncogenic role of hypoxia-inducible circWSB1 in the progression of BC, highlighting the tremendous possibility of circWSB1 to be a promising biomarker for BC.

To date, only several hypoxia-related circRNAs have been reported in tumorigenesis and progression of BC. For example, circZFR serves as a sponge of miR-578 to promote BC progression by modulating the expression of HIF1α [28]. Another circRNA named circRNF20 promotes tumorigenesis of BC and Warburg effect by miR-487a/HIF-1α/HK2 axis [29]. Furthermore, Zhan et al. demonstrated that circHIF1α from hypoxic CAFs (cancer-associated fibroblasts) exosomes may play an important role in BC cell stemness via sponging miR-580-5p [30]. These reports, together with our findings, indicated that circRNAs might play pivotal roles in the regulation of hypoxia-induced signaling cascades. However, almost all of these hypoxia-related circRNAs acted as miRNA sponges. In addition, circRNAs could also function as scaffolds facilitating the assembly of protein complexes, regulators of transcription and RBP-binding partners [31–33]. Even a minor fraction of circRNAs have been proved to be translated into proteins in a cap-independent translation initiation manner [34]. Although circRNAs exert their functions in multiple ways, the overwhelming majority of circRNAs were characterized to sponge miRNAs in cellular physiology. In the present study, we uncovered a distinct mechanism that hypoxia-induced circWSB1 could physically bind with deubiquitinase USP10 and abolished the interplay between USP10 and its target protein p53, thereby leading to the poly-ubiquitination and subsequent degradation of p53 protein under hypoxic conditions.

USP10 is an important member of the ubiquitin-specific protease family, which has been found to be widely expressed in both cytoplasm and nucleus of almost all cells. As a deubiquitinating enzyme, USP10 could reverse the ubiquitination of substrates through removing ubiquitin (Ub) molecules from the C-terminal of Ub-conjugated target proteins and maintain intracellular protein homeostasis by recycling Ub [35]. Accumulating evidence demonstrated that USP10 play crucial roles as a tumor-promoter or tumor-suppressor in the tumorigenesis and development of various cancers. For example, USP10 interacts with and stabilizes YAP/TAZ and SMAD4 to promote the proliferation of hepatocellular carcinoma [36, 37], while another study indicated that USP10 inhibited the poly-ubiquitylation of AMPKα and PTEN to suppress hepatocellular carcinoma progression [38]. Additionally, there are also conflicting reports in lung cancer, with findings pinpointing that USP10 enhanced the stability and activity of PTEN to inhibit non-small cell lung cancer cell proliferation [39], whereas another research suggesting that USP10 deubiquitinated oncogenic protein HDAC6 to confer cisplatin resistance of non-small cell lung cancer [40]. These studies revealed that the diverse roles of USP10 in cancers are due to its target proteins. The prior study indicated that inhibition of USP10 at both genetic and pharmacological levels effectively attenuated curcumin-induced paraptosis in BC cells [41]. However, the biological role and underlying mechanisms of USP10 in BC remains largely unclear.

By utilizing microarray and KEGG analyses, we found that p53 signaling pathway was significantly enriched after knockdown of circWSB1 in hypoxic MCF-7 cells. p53 has been widely considered as a tumor suppressor in human cancers, which functions as a transcription factor to suppress cell proliferation by triggering cell cycle arrest and apoptosis through transactivation of its target genes, such as p21 and BAX [42]. Further investigations revealed that circWSB1 could negatively regulate the protein level of p53 and its downstream p21 and Bax in hypoxic BC cells, suggesting that hypoxia-induced circWSB1 might exert its function through p53 signaling pathway. Previous study indicated that mild hypoxia could help tumor cells to survive through decreasing p53 protein level [43]. Our results demonstrated that circWSB1 might participate in the regulation of p53 by hypoxia. It has been reported that USP10 translocates to the nucleus under genotoxic stress and then binds with and deubiquitinates p53 protein through its N-terminal region (1-100aa), which inhibited the proliferation of tumor with wild-type p53 [20]. Mutations of *TP53* gene have been frequently observed in human cancers, however, more than 75% of BC cases are found to have wild-type of p53 [17]. And the two BC cell lines used in our study also express wild-type of p53 [44]. Consistent with previous study, we verified that USP10 could also bind with p53 through its N-terminal region (1-100aa) and deubiquitinate p53 in BC cells. Moreover, ectopic expression of USP10 significantly compromised the proliferation of BC cells and led to cell cycle arrest as well as apoptosis under hypoxia.

Therefore, we speculated that circWSB1 might regulate p53 signaling pathway through USP10, further promoting the proliferation of BC cells. Intriguingly, we found that the p53 binding domain of USP10, rather than its catalytic core domain or other domains, was also responsible for the interaction between circWSB1 and USP10, suggesting that circWSB1 and p53 might competitively bind with USP10. That is to say, the interaction between circWSB1 and USP10 could prevent the binding of USP10 to p53, resulting in the ubiquitination and degradation of p53. As anticipated, we found that ectopic expression of circWSB1 inhibited the binding of USP10 and p53. The rescue experiments *vitro* and in vivo further validated that circWSB1 could promote BC progression through destabilizing p53 by interacting with USP10. Our study provides the first evidence that circRNA might affect the stability of p53 through interacting with deubiquitinase USP10 under hypoxic conditions, which also reveals the diversity and complexity of circRNA functions.

## Conclusions

Taken together, we firstly discover that circWSB1 is dramatically induced by HIF1α under hypoxic conditions and could bind with USP10 to disrupt the interaction between USP10 and p53, which leads to the poly-ubiquitination and subsequent degradation of p53, thus assisting tumor cells to survive under hypoxia and promoting the progression of BC. Our findings might help to understand that how hypoxia contributes to the development of BC through circWSB1 and highlight the possibility that circWSB1 might be a novel prognostic marker and therapeutic target for BC.

## Supplementary Information

**Additional file 1:** **Table S1.** Sequences of the primers used in present study. Table S2. Sequences of siRNAs and shRNAs used in this study. Table S3. Correlation between circWSB1 expression and clinicopathological features of 288 BC patients.

**Additional file 2:** **Figure S1.** a Relative expression of HIF1α in 1100 BC tissues and 111 normal tissues of TCGA database. b Kaplan-Meier survival curve of these BC patients according to the expression of HIF1α. The patients were categorized into HIF1α low or high expression group by the media of HIF1α level. c Volcano plot of the differentially expressed mRNAs between MCF-7 cells treated with normoxia and hypoxia for 48 h. The blue dots and red dots represent downregulated and upregulated mRNAs with statistical significance, respectively. d KEGG pathway analysis of the differentially expressed mRNAs as per (c).

**Additional file 3:** **Figure S2.** qRT-PCR analyses to validate the efficiency of the overexpression vector and siRNAs of HIF1α in MCF-7 cells. Data were showed as mean ± SD, ***P<0.001.

**Additional file 4:** **Figure S3.** a qRT-PCR analyses of circWSB1expression in MCF-7 and MDA-MB-453 cells transfected with indicated vectors. b and c The mRNA (b) and protein (c) levels of WSB1 were determined by qRT-PCR and western blot, respectively. d and e CCK-8 (d) and colony formation (e) assays were conducted in MCF-7 and MDA-MB-453 cells after overexpression of circWSB1. f Western blot analyses of MCF-7 and MDA-MB-453 cells after upregulation of circWSB1 with indicated antibodies. g The expression level of circWSB1 in stable MCF-7 cells was examined by qRT-PCR. Data were showed as mean ± SD, ns, no significance. **P<0.01, ***P<0.001.

**Additional file 5:** **Figure S4.** a and b The effects of circWSB1 on the expression level of USP10 in MCF-7 and MDA-MB-453 cells. c The effect of USP10 on the expression of circWSB1 in MCF-7 and MDA-MB-453 cells. Data were showed as mean ± SD, ns, no significance.

**Additional file 6:** **Figure S5.** a Western blot analyses of MCF-7 and MDA-MB-453 cells after overexpression of USP10. b and c CCK-8 (b) and colony formation assays (c) were conducted in MCF-7 and MDA-MB-453 cells after ectopic expression of USP10. Data were showed as mean ± SD, *P<0.05, **P<0.01, ***P<0.001.

**Additional file 7:** **Figure S6.** a Western blot analyses of MDA-MB-453 cells after transfected with indicated siRNAs under hypoxia. b CCK-8 assays performed in MDA-MB-453 cells after co-silencing of circWSB1 and USP10 under hypoxic conditions. c Colony formation assays of MCF-7 and MDA-MB-453 cells after co-overexpression or co-knockdown of circWSB1 and USP10 under hypoxia. d-e Flow cytometric cell cycle assays (d) and apoptosis analyses (e) of hypoxic MDA-MB-453 cells treated with indicated siRNAs. Data were showed as mean ± SD, *P<0.05, **P<0.01, ***P<0.001.

## Data Availability

The datasets used during the current study are available from the corresponding author on reasonable request.

## Abbreviations

BC: Breast cancer
HIF1: Hypoxia-inducible factor 1
HREs: Hypoxia response elements
CircRNA: Circular RNA
WSB1: WD repeat and SOCS box containing 1
USP10: Ubiquitin specific peptidase 10
TCGA: The Cancer Genome Atlas
qRT-PCR: Quantitative real-time PCR
ISH: In situ hybridization
FISH: Fluorescence in situ hybridization
IF: Immunofluorescence
ChIP: Chromatin immunoprecipitation
RIP: RNA immunoprecipitation
Co-IP: Co-immunoprecipitation
RT-PCR: Reverse transcription PCR
