# Supplementary material for: Hypoxia-induced circWSB1 promotes breast cancer progression through destabilizing p53 by interacting with USP10
Source: Mol Cancer. 2022 Mar 29;21:88. doi: 10.1186/s12943-022-01567-z (PMC8961958; doi:10.1186/s12943-022-01567-z)
Supplement: Supplementary file 1 — Additional file 1: Table S1. Sequences of the primers used in present study. Table S2. Sequences of siRNAs and shRNAs used in this study. Table S3. Correlation between circWSB1 expression and clinicopathological features of 288 BC patients. [file 12943_2022_1567_MOESM1_ESM.docx]

**Table S1. Sequences of primers used in this study.**

| Gene | Primer sequences |
| --- | --- |
| circWSB1 (Divergent) | F: 5’-ATTCTGATGGAATTTGGTCTCT-3’ |
|  | R: 5’-CAAAAGCAAGACTCCAGACTAT-3’ |
| circWSB1 (Convergent) | F: 5’-AGCTTGATCCTGGTGTCAGC-3’ |
|  | R: 5’-ACTGCTTTACTGGCTCCGAC-3’ |
| hsa_circ_0042489 | F: 5’- ACATTCTGATGGAATTTGGTGAG -3’ |
|  | R: 5’- TTACTGTGCGATGTCCTTGT -3’ |
| hsa_circ_0042493 | F: 5’- TGGGACCTGAAAGATGATGT -3’ |
|  | R: 5’- GCAAGACTCCAGACTATATCTC -3’ |
| hsa_circ_0042496 | F: 5’- GCAAGCCTTGCTGATGATAAT -3’ |
|  | R: 5’- AGCAAGACTCCAGACTATATCTC -3’ |
| GAPDH | F: 5’-GAAGGTGAAGGTCGGAGTC-3’ |
|  | R: 5’-GAAGATGGTGATGGGATTTC-3’ |
| HIF1α | F: 5’-GAACGTCGAAAAGAAAAGTCTCG-3’ |
|  | R: 5’- CCTTATCAAGATGCGAACTCACA -3’ |
| β-actin (Convergent) | F: 5’-CATGTACGTTGCTATCCAGGC-3’ |
|  | R: 5’-CTCCTTAATGTCACGCACGAT-3’ |
| β-actin (Divergent) | F: 5’-AAATCGTGCGTGACATTAAGGAGA-3’ |
|  | R: 5’-CATACCCCTCGTAGATGGGCA-3’ |
| WSB1 | F: 5’-AGCTTGATCCTGGTGTCAGC-3’ |
|  | R: 5’-ACTGCTTTACTGGCTCCGAC-3’ |
| U6 | F: 5’-CTCGCTTCGGCAGCACA-3’ |
|  | R: 5’-AACGCTTCACGAATTTGCGT-3’ |
|  | RT: 5’-CTCGCTTCGGCAGCACAPCR-3’ |
| WSB1 promoter P1 | F: 5’-TGGTGGCTCACGTTGTAATC-3’ |
|  | R: 5’-AGGCTAGTCTCGAACTCCTG-3’ |
| WSB1 promoter P3 | F: 5’-GAGATCACGTCCCGTGC-3’ |
|  | R: 5’-GGCAGGGGCGCGG-3’ |

**Table S2. Sequences of siRNAs and shRNAs used in this study.**

| Definition | sequences |
| --- | --- |
| si-NC | 5’-TTCTCCGAACGTGTCACGTAA-3’ |
| si-circ#1 | 5’-TGGAATTTGGTCTCTTGCA-3’ |
| si-circ#2 | 5’-GAATTTGGTCTCTTGCATG-3’ |
| sh-NC | 5’- TTCTCCGAACGTGTCACGTAATTCAAGAGATTACGTGACACGTTCGGAGAATTTTTTC -3’ |
| sh-circ#1 | 5’- TGGAATTTGGTCTCTTGCATGTTCAAGAGACATGCAAGAGACCAAATTCCATTTTTTG-3’ |
| si-USP10#1 | 5’-CCTCGCTTTGGATGGAAGTTCTAAT-3’ |
| si-USP10#2 | 5’-CAGTGACATTGTGCCTGACAGTCCT-3’ |
| sh-USP10#1 | 5’-GATCCGCCTCGCTTTGGATGGAAGTTCTAATTTCAAGAGAATTAGAACTTCCATCCAAAGCGAGGTTTTTTG-3’ |

**Table S3** Correlation between circWSB1 expression and clinicopathological features in 288 BC patients.

| Characteristics | | circWSB1 | | Chi-square | P value |
| --- | --- | --- | --- | --- | --- |
|  |  | Low | High |  |  |
| Age | <50 | 63 | 49 | 0.000 | 1.000 |
|  | ≥50 | 99 | 77 |  |  |
| Grade | I-II | 141 | 102 | 1.990 | 0.158 |
|  | III | 21 | 24 |  |  |
| T stage | T1 | 53 | 36 | 2.006 | 0.157 |
|  | T2-3 | 108 | 89 |  |  |
| N stage | N0 | 70 | 60 | 0.605 | 0.437 |
|  | N1-3 | 90 | 64 |  |  |
| TNM stage | I-II | 112 | 80 | 0.784 | 0.376 |
|  | III | 48 | 43 |  |  |
