# Supplementary figures and images for: Hypoxia-induced circWSB1 promotes breast cancer progression through destabilizing p53 by interacting with USP10
Source: Mol Cancer. 2022 Mar 29;21:88. doi: 10.1186/s12943-022-01567-z (PMC8961958; doi:10.1186/s12943-022-01567-z)

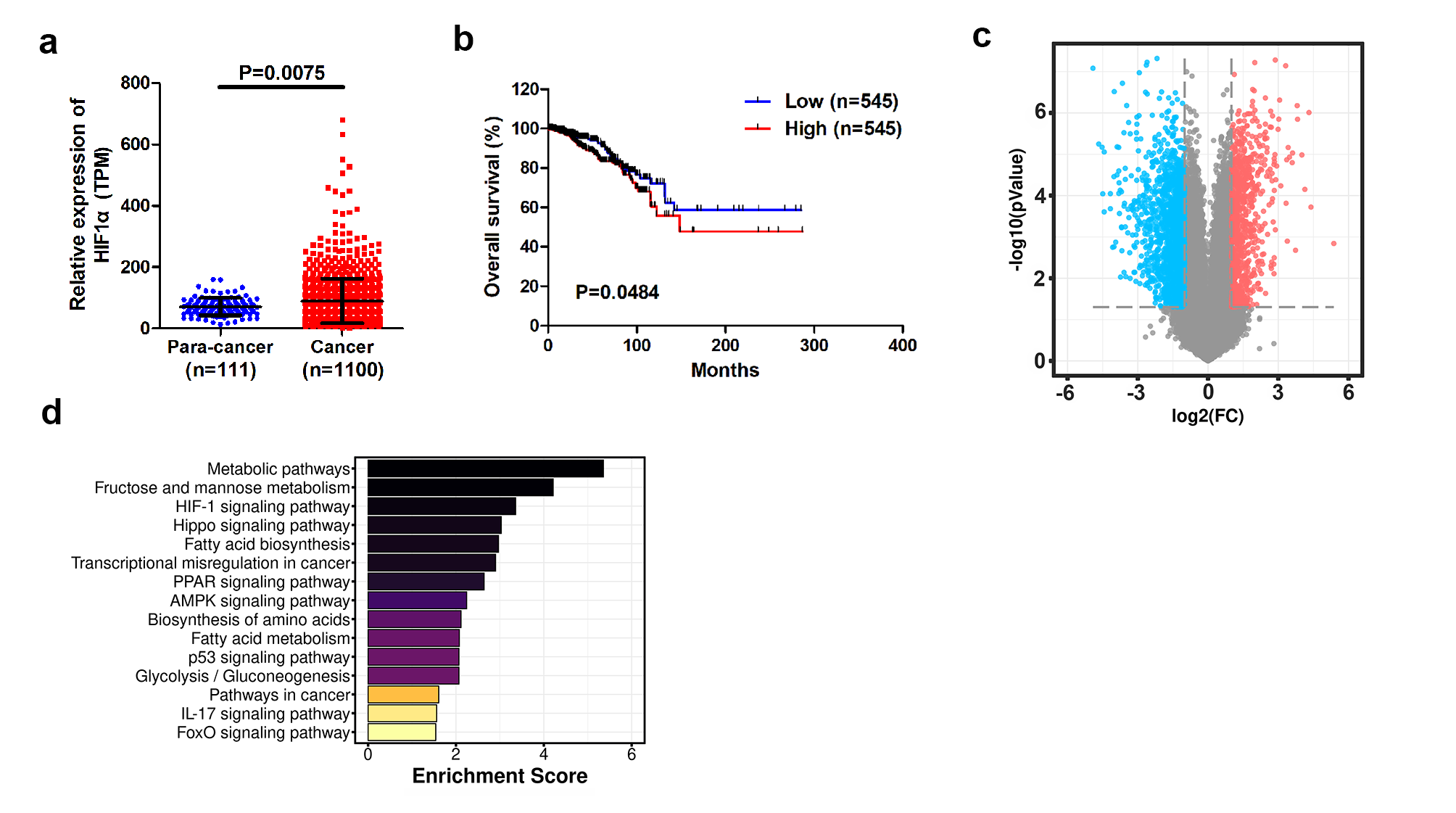

Supplement: Supplementary file 2 — Additional file 2: Figure S1. a Relative expression of HIF1α in 1100 BC tissues and 111 normal tissues of TCGA database. b Kaplan-Meier survival curve of these BC patients according to the expression of HIF1α. The patients were categorized into HIF1α low or high expression group by the media of HIF1α level. c Volcano plot of the differentially expressed mRNAs between MCF-7 cells treated with normoxia and hypoxia for 48 h. The blue dots and red dots represent downregulated and upregulated mRNAs with statistical significance, respectively. d KEGG pathway analysis of the differentially expressed mRNAs as per (c). [file 12943_2022_1567_MOESM2_ESM.tif]

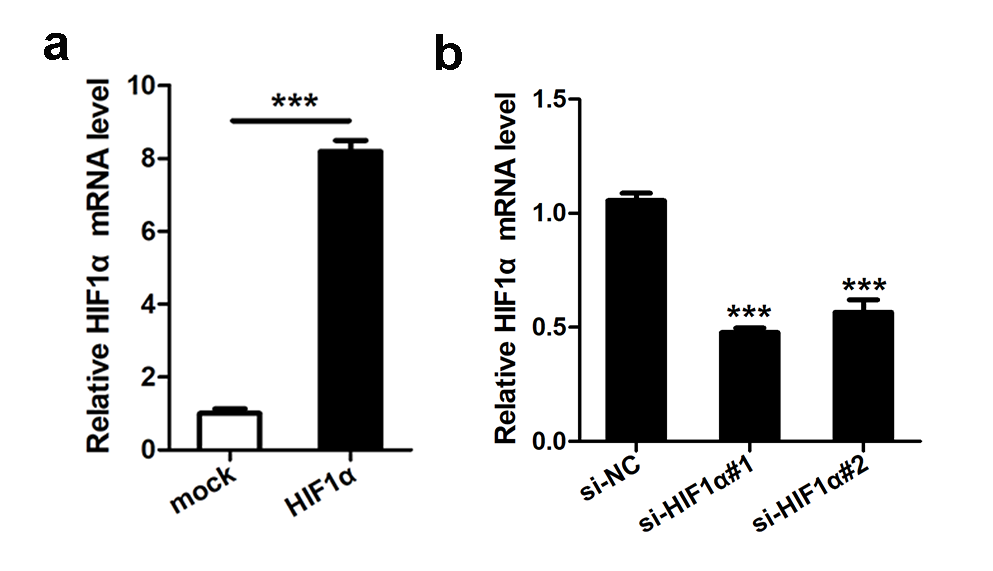

Supplement: Supplementary file 3 — Additional file 3: Figure S2. qRT-PCR analyses to validate the efficiency of the overexpression vector and siRNAs of HIF1α in MCF-7 cells. Data were showed as mean ± SD, ***P<0.001. [file 12943_2022_1567_MOESM3_ESM.tif]

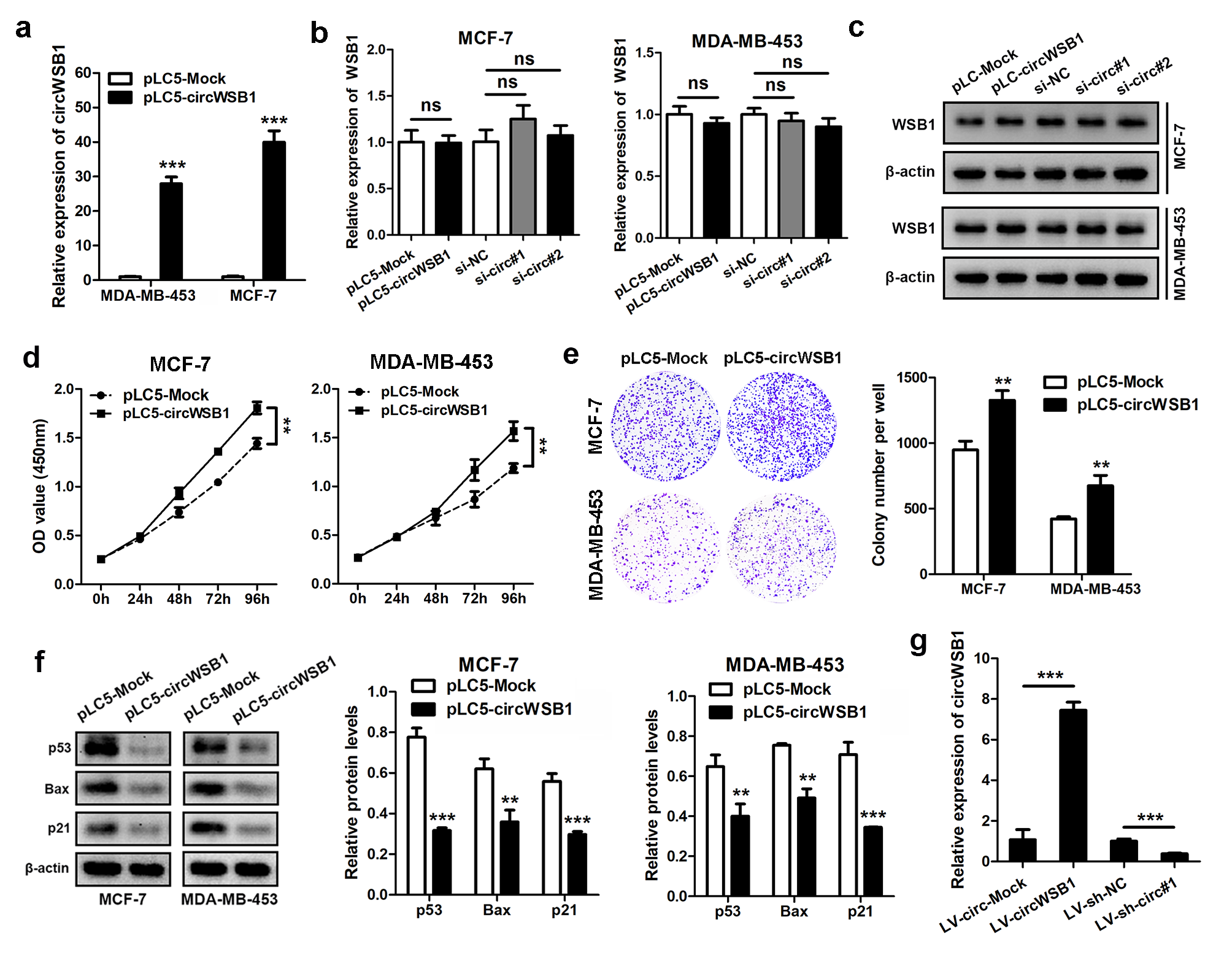

Supplement: Supplementary file 4 — Additional file 4: Figure S3. a qRT-PCR analyses of circWSB1expression in MCF-7 and MDA-MB-453 cells transfected with indicated vectors. b and c The mRNA (b) and protein (c) levels of WSB1 were determined by qRT-PCR and western blot, respectively. d and e CCK-8 (d) and colony formation (e) assays were conducted in MCF-7 and MDA-MB-453 cells after overexpression of circWSB1. f Western blot analyses of MCF-7 and MDA-MB-453 cells after upregulation of circWSB1 with indicated antibodies. g The expression level of circWSB1 in stable MCF-7 cells was examined by qRT-PCR. Data were showed as mean ± SD, ns, no significance. **P<0.01, ***P<0.001. [file 12943_2022_1567_MOESM4_ESM.tif]

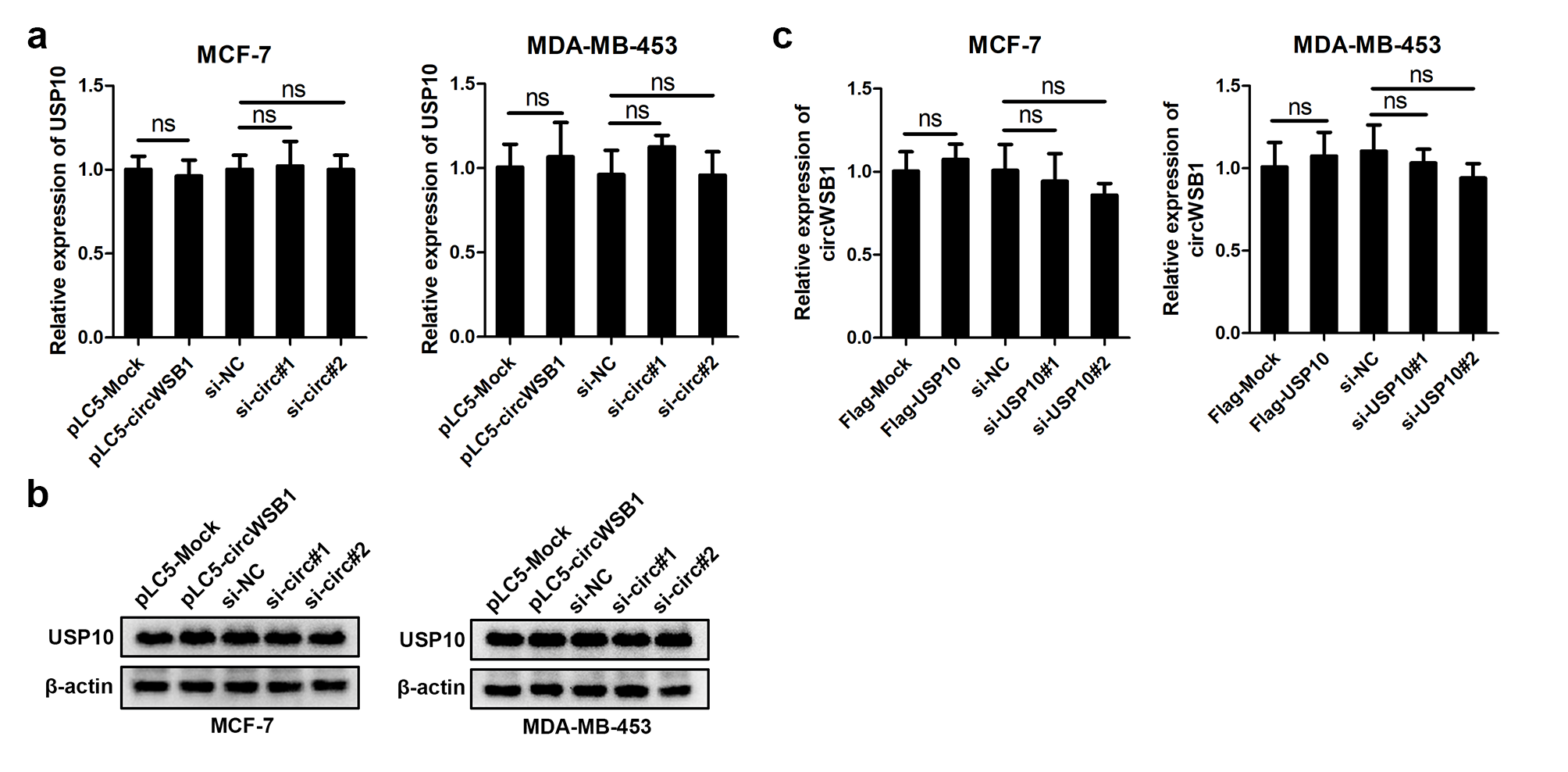

Supplement: Supplementary file 5 — Additional file 5: Figure S4. a and b The effects of circWSB1 on the expression level of USP10 in MCF-7 and MDA-MB-453 cells. c The effect of USP10 on the expression of circWSB1 in MCF-7 and MDA-MB-453 cells. Data were showed as mean ± SD, ns, no significance. [file 12943_2022_1567_MOESM5_ESM.tif]

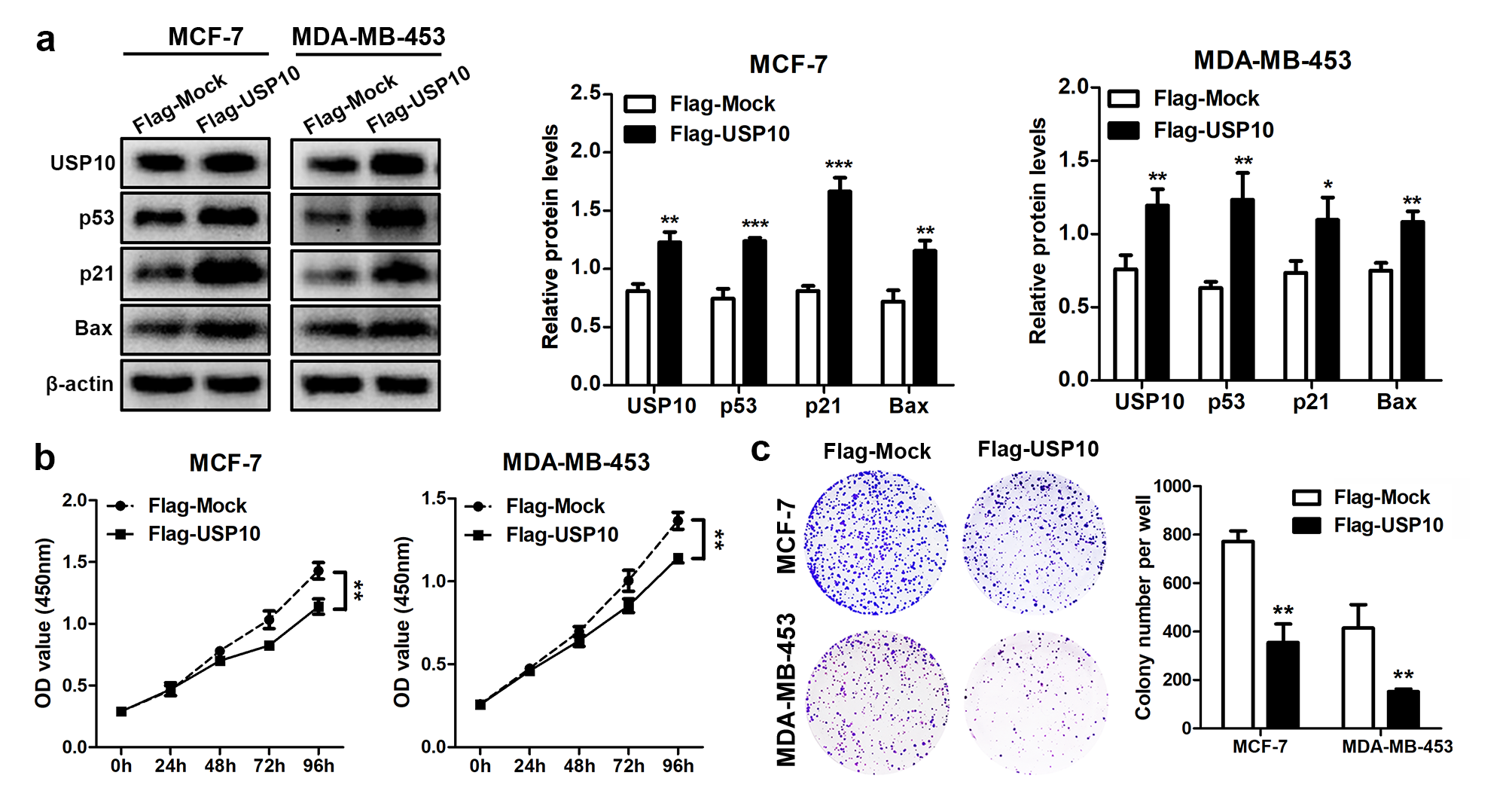

Supplement: Supplementary file 6 — Additional file 6: Figure S5. a Western blot analyses of MCF-7 and MDA-MB-453 cells after overexpression of USP10. b and c CCK-8 (b) and colony formation assays (c) were conducted in MCF-7 and MDA-MB-453 cells after ectopic expression of USP10. Data were showed as mean ± SD, *P<0.05, **P<0.01, ***P<0.001. [file 12943_2022_1567_MOESM6_ESM.tif]

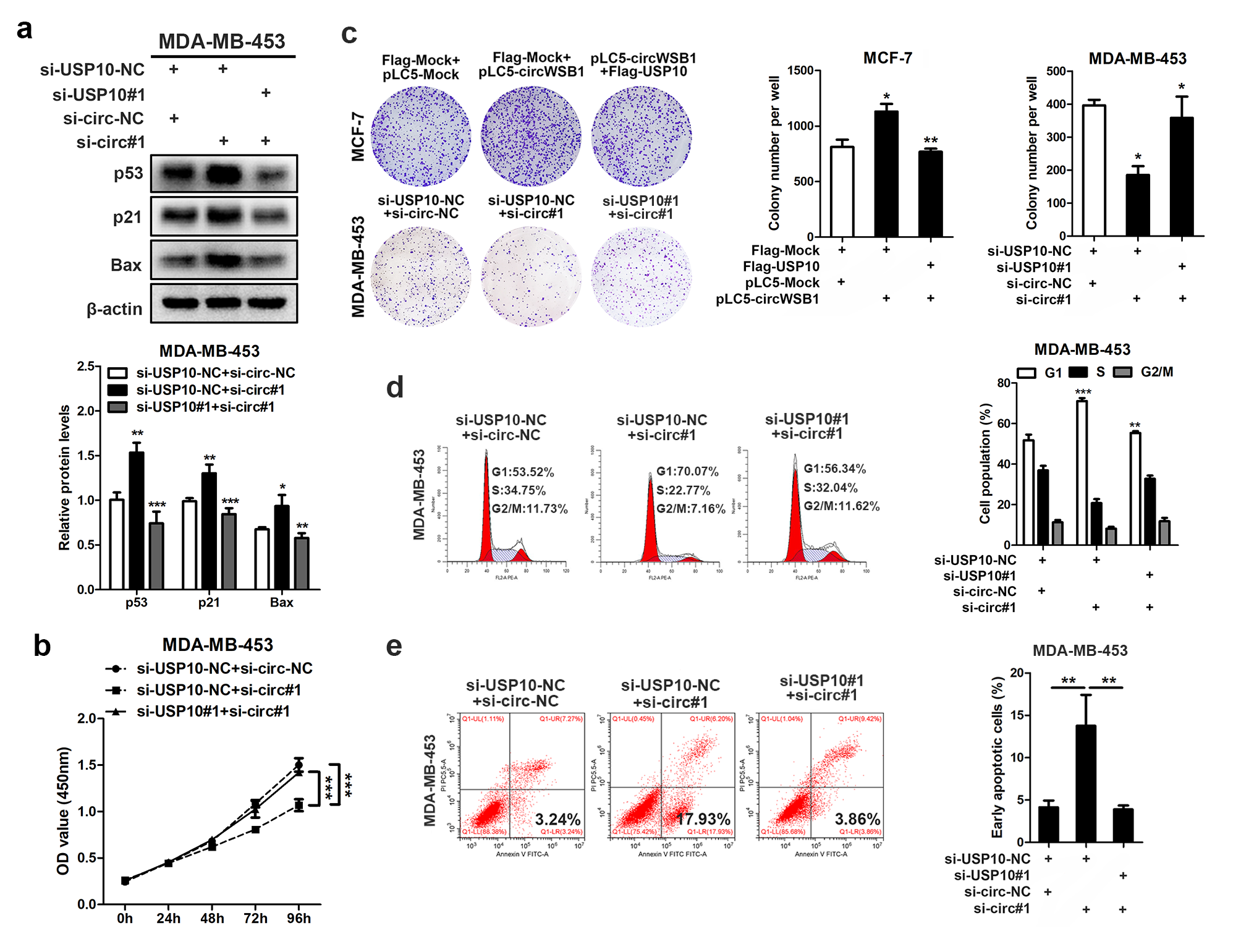

Supplement: Supplementary file 7 — Additional file 7: Figure S6. a Western blot analyses of MDA-MB-453 cells after transfected with indicated siRNAs under hypoxia. b CCK-8 assays performed in MDA-MB-453 cells after co-silencing of circWSB1 and USP10 under hypoxic conditions. c Colony formation assays of MCF-7 and MDA-MB-453 cells after co-overexpression or co-knockdown of circWSB1 and USP10 under hypoxia. d-e Flow cytometric cell cycle assays (d) and apoptosis analyses (e) of hypoxic MDA-MB-453 cells treated with indicated siRNAs. Data were showed as mean ± SD, *P<0.05, **P<0.01, ***P<0.001. [file 12943_2022_1567_MOESM7_ESM.tif]
